# Prevalence and environmental abundance of the TSET complex in cosmopolitan algal groups

**DOI:** 10.1016/j.isci.2025.112679

**Published:** 2025-05-15

**Authors:** Mathias Penot-Raquin, Mandeep Sivia, Kelly M. Fafoumi, Raegan Larson, Richard G. Dorrell, Joel B. Dacks

**Affiliations:** 1 Institut de Biologie de l’École Normale Supérieure (IBENS), École Normale Supérieure, CNRS, INSERM, PSL Université Paris, 75005 Paris, France; 2 Research Federation for the study of Global Ocean Systems Ecology and Evolution, FR2022/Tara Oceans, 75016 Paris, France; 3 Sorbonne Université, CNRS, IBPS, UMR 7238, Computational, Quantitative and Synthetic Biology (CQSB), 75005 Paris, France; 4 Division of Infectious Diseases, Department of Medicine, and Department of Biological Sciences University of Alberta, 1-124 Clinical Sciences Building, 11350-83 Avenue, Edmonton, Alberta T6G 2G3, Canada; 5 Centre for Life’s Origins and Evolution, Department of Genetics, Evolution and the Environment, University College London, Darwin Building, Gower Street, London WC1E 6BT, UK

**Keywords:** Cell biology, Plant biology, Plant evolution, Aquatic biology, Plant development

## Abstract

Classical cell biology paradigms are largely established on animal, fungal, and plant models which constitute a small fraction of eukaryotic diversity. Some important cellular machinery has been historically overlooked due to their absence from animals and fungi, e.g., the membrane-trafficking complex TSET involved in plant cell division and endocytosis. Here, we document TSET complexes in distantly related photosynthetic eukaryotic groups (green algae, red algae, haptophytes, cryptophytes, and stramenopiles including diatoms). 3D modeling predicts that at least some stramenopile-encoded subunits share conserved structural features with plant orthologues, and gene expression analysis from the diatom *Phaeodactylum tricornutum* shows that they are co-expressed with endomembrane trafficking proteins. Finally, diatom TSET genes are detectable in *meta*-transcriptomic data from *Tara* Oceans, suggesting functional roles in the wild. These results support the importance of integrating non-model organisms into our understanding of eukaryotic cell biology, as they may reveal underappreciated protein complexes essential for cellular and ecosystem functions.

## Highlights

• TSET is key to plant endocytosis but absent in other models, undermining its generality
• TSET is present in distant eukaryotic groups and expressed by diatoms worldwide
• Plant and algal TSET are predicted to share structural and functional homologies
• TSET should be included as a trafficking component for general cellular models

## Graphical abstract

**Figure:**
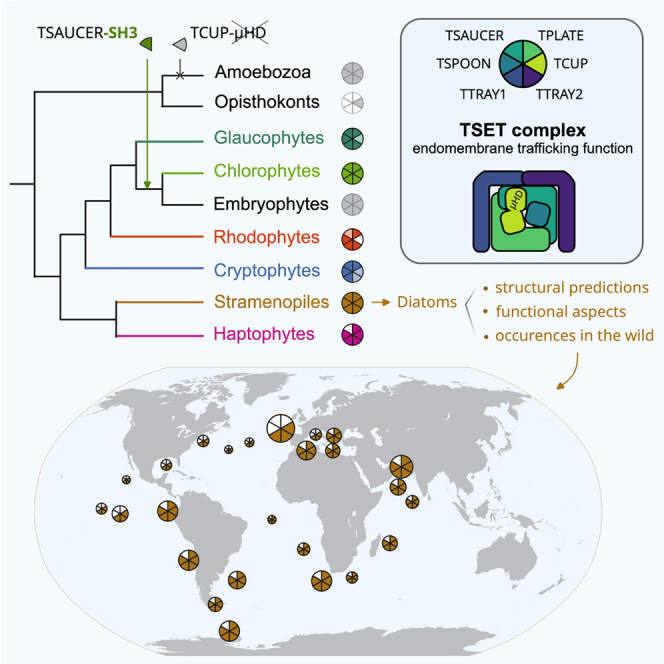

## Introduction

Most eukaryotic diversity relates to microscopic, i.e., protist, lineages,^1^ whose taxonomic breadth is being progressively revealed through next-generation genome and transcriptome sequencing projects.^1^^,^^2^ Recent advances in understanding the eukaryotic tree include the identification of new phylum- and kingdom-level protist lineages,^3^^,^^4^ and phylogenomic assembly of all eukaryotes into eleven “supergroups”.^1^ Nonetheless, much of our understanding of eukaryotic cell biology is still defined by model organisms from two supergroups. These are the Opisthokonta, encompassing animals and fungi, and to a lesser extent the Archaeplastida,^5^ including plants and green and red algae. The biology of protists and algae in the other supergroups (Amoebozoa, CRuMs, Provora, Hemimastigophora, Metamonada, Discoba, Cryptista, Haptista, and TSAR) has been comparatively understudied, despite their massive medical, biotechnological, agricultural, and environmental relevance (reviewed in^1^). It is imperative that our understanding of cellular biology also encompasses these organisms, as they have the potential to transform, and challenge, our view of how eukaryotic cells conventionally function.^6^

With a few key exceptions (e.g., parasitic species^7^^,^^8^^,^^9^), protist cell biology has remained underexplored, reflecting the historical inaccessibility, uncultivability, and intractability to laboratory manipulation of many protist groups.^10^ However, technical advances are erasing these barriers. Environmental sequencing has expanded our understanding of protist diversity in the wild,^11^ and is allowing us identification of novel uncultivated eukaryotic groups, typically from 18S rDNA *meta*-barcoding.^12^ The progressive domestication of protist groups in laboratory culture, which may involve overcoming cultivation roadblocks,^10^^,^^13^^,^^14^ allows us to understand their functional genomic diversity, gene expression, and proteomes. Concerted efforts to develop genetic tools for cultured protists is expanding and rebalancing our view of eukaryotic cell biology,^15^ termed by some authors a “cultural renaissance”.^16^ These combined techniques have revealed an intriguing set of cellular factors, termed jötnarlogs,^17^ which are homologues with “the evolutionary pattern of being ancient but hidden from view”, as with the walled-off ancient world of Jotunheim in Norse mythology. Jötnarlogs are genes not detected in yeast or animal systems, hence hidden from classical cell biology models. These genes, however, are found in sufficiently diverse eukaryotic supergroups to infer their presence in the LECA (*Last Eukaryotic Common Ancestor*).^17^ Within just the membrane-trafficking system there are at least 12 examples.^17^^,^^18^ While the existence of jötnarlogs is well-established, their taxonomic prevalence in environmentally and medically important protists is poorly understood, and their overall significance to eukaryotic cell function remains largely unproven.

One of the best studied examples of a jötnarlog is the adaptin-related membrane trafficking complex, TSET, originally identified as the “TPC” complex in the model plant *Arabidopsis thaliana*,^19^ followed by its identification and characterization in the distantly related amoebozoan *Dictyostelium discoideum*.^20^ TSET comprises at least six subunits (TSPOON, TCUP, TPLATE, TSAUCER, TTRAY1, and TTRAY2), homologous to, but distinct from, the five conventional adaptins and the COPI complexes that serve as cargo-recognition machinery in vesicle formation across the endomembrane system.^21^ In plants, TSET plays roles in phragmoplast deposition and cell division,^22^ actin-mediated autophagy,^23^ and clathrin-mediated endocytosis alongside the conventional adaptor complex AP2.^19^^,^^24^ In *D. discoideum* it likewise has endocytotic functions, although potentially without clathrin involvement.^20^ This contrasts with Opisthokonta^19^^,^^20^ models that retain only a protein derived from TCUP (FCHO in animals and Syp1 in yeast), which nonetheless has a role as part of an accessory protein complex (FEI complex) that plays a role in cargo sorting at the plasma membrane in clathrin-mediated endocytosis,^25^ as well as ESCRT0 mediated sorting of ubiquitinated cargo to the multi-vesicular body.^26^

An initial comparative genomic evaluation of TSET, published in 2014, reported its presence across the eukaryotes,^20^ and most likely presence in LECA, although with varying levels of conservation. This included eukaryotic algae, e.g., green and red algae (in Archaeplastida), cryptophytes (in Cryptista), haptophytes (in Haptista), and stramenopiles (including diatoms, in TSAR) that are distantly positioned across the eukaryotic tree (Figure S1). These groups represent a majority of total algal abundance, e.g., considering metagenome data from the *Tara* Oceans campaign,^10^^,^^11^ performing 30–40% of planetary primary production.^10^^,^^11^^,^^27^ Many of them exhibit rapid turnover and short generation times in blooming cycles,^28^ for which roles for TSET in cell division may be relevant. Moreover, many of these groups use phagotrophic strategies alongside photosynthesis (i.e., photo-mixotrophy),^29^ and several of them include fully heterotrophic members (e.g., the plant pathogens oomycetes, and the hyper-abundant human gut commensal *Blastocystis hominis* in the stramenopiles), for which the endocytotic functions of TSET may equally be relevant. In the initial TSET pan-eukaryotic survey,^20^ entire complexes were detected in two green algal species, but incomplete complexes (fewer than three of six subunits) were found in the four stramenopile genomes sampled. Complete TSET complexes have since been annotated in the mixotrophic green alga *Cymbomonas tetramitiformis*,^30^ and in *B. hominis* and its close relative *Proteromonas lacerate*.^31^ To date, in contrast, only single TSET subunits have been detected in glaucophytes, haptophytes, and cryptophytes, and none have been found in red algae, rendering its overall conservation and functional relevance to eukaryotic algae uncertain.^20^^,^^32^

Here, we revisit the question of TSET prevalence across eukaryotic algae, using an iterative homologue retrieval (AMOEBAE) approach,^33^ and phylogenetic validation. We further use three-dimensional (3D) modeling to assess the potential conservation of TSET structure, and evaluate gene expression data from the model diatom *Phaeodactylum tricornutum*, and from *Tara* Ocean *meta*-transcriptomes, to hypothesize functional roles for TSET in these groups. Our study establishes the relevance of TSET to algal biology, and underlines the importance of cultivated and uncultured microbes for understanding fundamental cell processes across the eukaryotic Tree of Life.

### Results

#### Comparative genome detection of widespread TSET occurrence in eukaryotic algae

To establish the prevalence of TSET in algae and their relatives, we assembled a dataset of 742 genomes and transcriptomes for five groups (green algae including nine glaucophytes - 281 libraries; stramenopiles - 335 libraries; haptophytes - 50 libraries; red algae - 42 libraries; cryptophytes - 34 libraries; Data S1). This approach was selected for assessment of orthologues in as many lineages as possible, prioritizing maximum taxonomic representation for breadth and depth of representation (high sensitivity) over quantitative assessment of prevalence in high quality references (low false negative error rates). Thus, below, we do not draw conclusions from failures to identify candidates in individual species and instead derive patterns based on larger taxonomic groupings. We used the presence of subunits from the AP1 complex as a positive control for search efficacy, since AP1 is believed to be almost universally conserved in eukaryotes.^17^

**Figure 1:**
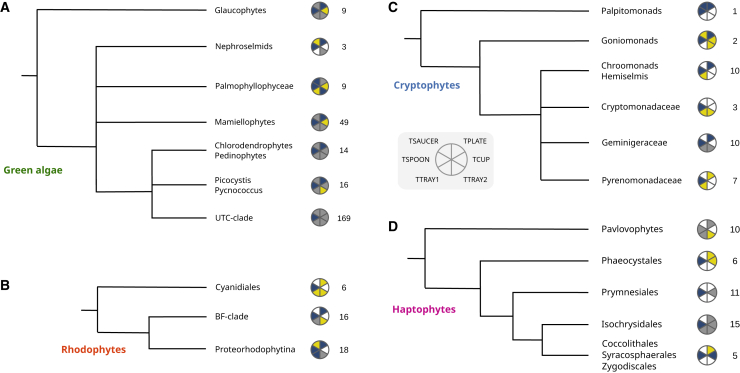
Prevalence of TSET in major marine algal groups Illustration of TSET subunit detection in the subgroups of each of four algal groups. Yellow sectors indicate that an orthologue was detected in a single dataset. Gray sectors indicate that orthologues were detected in fewer than 50% of the datasets. Blue sectors indicate that orthologues were detected in ≥50% of the datasets examined. The number of AP1-positive genomes/transcriptome libraries inspected is indicated on the right side of the corresponding group. Branching orders are schematics based on existing taxonomic knowledge for each group, per.^1^ (A) Green algae and glaucophytes. (B) Red Algae. (C) Cryptophytes. (D) Haptophytes.

We began with an assessment of TSET in green algae. Extending from previous identification of full TSET complexes in land plants and the core Chlorophyceae *Chlamydomonas reinhardtii*, *Volvox carterae*, and *C. tetramitiformis*,^20^^,^^30^ we were able to identify each of the six TSET subunits in five chlorophyte groups studied, with only the Nephroselmids (for which only three transcriptomes were available) lacking identifiable TCUP and TTRAY1 (Figure 1A, Data S2). There were 17 instances of the complete TSET complex being identified within a given library, 14 of which were from the core Ulvophyceae-Trebouxiophyceae- Chlorophyceae (UTC clade), which can represent dominant green algae, particularly in coastal regions and in freshwater.^34^ Two further libraries with complete TSET complexes were from the Chlorodendrophytes-Pedinophytes that are sister to the UTC clade, and one from a distantly related Mamiellophyte (*Pyramimonas parkeae* CCMP726). TCUP was the least frequently detected subunit across this green algae dataset, only being found in 33/329 (12%) of individual libraries in which at least two AP1 subunits were found.

Related to the green algae and embryophytes are the red algae and glaucophytes within the supergroup Archaeplastida and (most likely) the cryptophytes within the Cryptista. Despite the comparatively small sampling of glaucophyte libraries (Figures 1A and Data S2), we were able to detect every TSET subunit, albeit with only one TCUP and no library possessing a complete complex. We observed an overall paucity of TSET subunits in the red algae, with no detected TCUP subunits and only one TSAUCER subunit (Figure 1B). Nearly the same degree of reduction was observed in the cryptophytes, for which none of the 33 AP1-positive libraries possessed a complete TSET and in which only a single TCUP subunit was found (Figure 1C and Data S2).

Haptophytes, which have previously been found to retain TCUP (e.g., in the model species *Emiliania huxleyi*^32^), were globally found to have all the subunits but lack TSAUCER (Figures 1D and Data S2). As per the situation for cryptophytes, no complete TSET complexes were detected across 47 AP1-positive haptophyte libraries, including multiple assemblies of the *E. huxleyi* genome and transcriptome.^35^ This suggests that this group does not have a complete TSET complex. However, TSPOON was detected in 30/47 of the studied haptophyte libraries (64%; Data S2), and its position within the TSPOON family, rather than with APsigma subunits, was confirmed by phylogenetic analysis (IQ-TREE) with 96% aLRT branch support (Figure S2).

**Figure 2:**
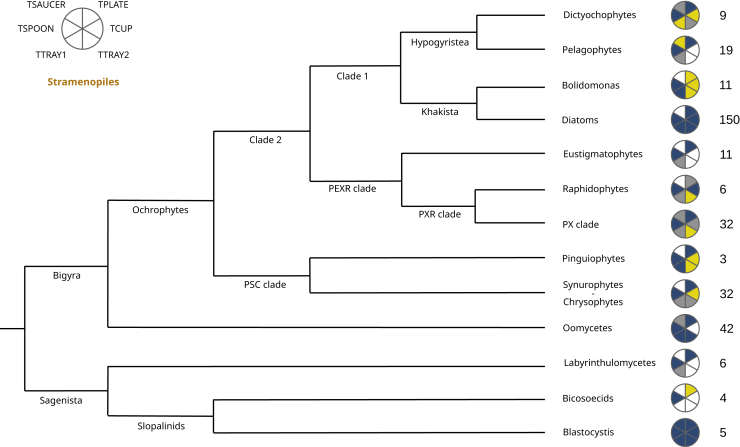
Distribution of TSET across stramenopiles Illustration of TSET subunit detection in the stramenopiles. Yellow sectors indicate that an orthologue was detected in a single dataset. Gray sectors indicate that orthologues were detected in fewer than 50% of the datasets. Blue sectors indicate that orthologues were detected in ≥50% of the datasets examined. The number of AP1-positive genomes/transcriptome libraries inspected is indicated on the right side of the corresponding group. Branching orders are schematics based on existing taxonomic knowledge for each group, per.^1^

Finally, for stramenopiles (335 libraries total), we identified all six components of the TSET complex in three of 13 taxonomic groups (dictyochophytes, the “PX” clade (Phaeophytes and Xanthophytes) and in *Blastocystis)*, and five of the six TSET subunits in six further taxonomic groups (*Bolidomonas*, diatoms, raphidophytes, pinguiophytes, synurophytes/chrysophytes, and oomycetes; Figure 2, Data S2). Beyond the complete *B. hominis* TSET already described,^31^ no individual stramenopile library possessed all six subunits. Most AP1-positive stramenopile libraries and all stramenopile taxonomic groups possessed detectable TPLATE (266/330 libraries, 81%) and TSPOON (259 libraries, 78%) subunits. TSAUCER was detected more rarely (19 libraries, 6%), with only a single validated subunit found in two taxonomic groups (pelagophytes, and dictyochophytes). Using the first dictyochophyte TSAUCER homologue detected (from *Dictyocha speculum* CCMP1381- MMETSP1174) as a search query into dictyophyte libraries, we were able to identify two additional homologues in *Pseudopedinella elastica* CCMP716 (combined MMETSP transcriptome) and *Rhizochromulina marina* CCMP1243 (single MMETSP transcriptome, MMETSP1173) (Data S2), although we were unable to detect these homologues in a similar search using the *Blastocystis* sequence as a query, suggesting that the detection of TSET homologues in our data may be limited by overall sequence conservation. Finally, individual phylogenies of each TSET subunit broadly recovered the same branching relationships as the species topology, suggesting largely vertical inheritance (Figure S2). Notwithstanding probable incomplete TSET homologue retrieval in individual stramenopile libraries, our data are most consistent with presence of a complete TSET in the stramenopile common ancestor.

For user inspection, all raw data files (AMOEBAE search results, alignments, trees) are provided in Data S3.

### Structural modeling shows conservation between stramenopile and plant TSET models

The comparative genomic and homology searching data that we obtained are consistent with widespread, if in some cases sporadic, distribution of TSET across eukaryotic algae and their non-photosynthetic relatives. However, this does not speak to TSET function in those groups. To do so, we took a structural modeling approach to assess possible structural homology, and subsequently a gene co-regulation approach.

To assess the general structural conservation of the TSET orthologues, AlphaFold^36^ was first used to generate individual structural models of all identified subunits (Data S4). It was then used to compare^36^^,^^37^ sequences from *A. thaliana* to those of *D. discoideum* that have been experimentally validated to perform similar roles in membrane-trafficking.^19^^,^^20^ The TSPOON, TSAUCER, and TPLATE subunit comparisons gave moderate TM scores (0.60, 0.50, and 0.47 respectively; Data S4) commonly used for assigning significant structural similarity.^37^ Weaker scores were observed for TTRAY1 (0.16) and TCUP (0.13; Data S4D and S4E). No direct comparison could be made for TTRAY2 as the TTRAY2 model could not be computed for *A. thaliana* (Data S4F). PAE plots of individual structural models are given in Data S4G.

**Figure 3:**
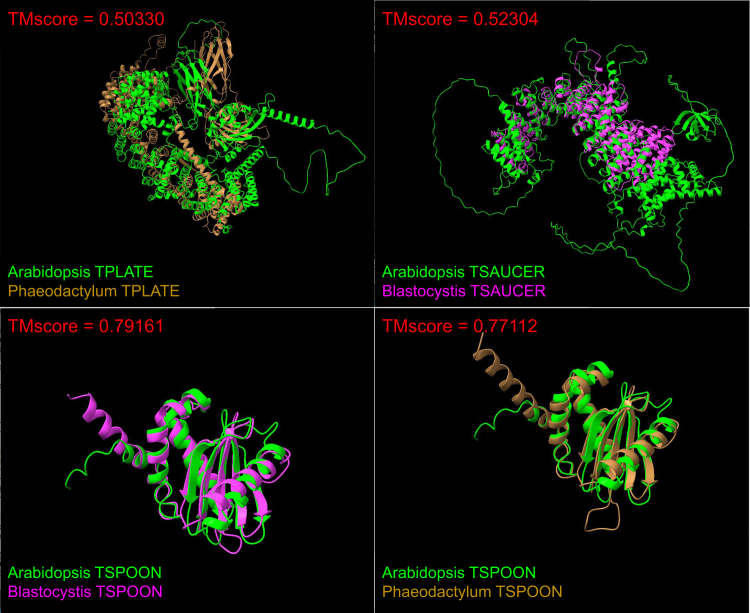
Modeling structural conservation between stramenopile and *A. thaliana* orthologues This figure shows superimposed AlphaFold predicted structures of TSET subunits, with the TM align score of >0.5 indicating structural conservation between the compared subunits. In all cases, taxa are colored as inset.

Next, AlphaFold predictions of the TSET subunits from two stramenopiles (*Blastocystis* sp., *P. tricornutum*) were compared to the *A. thaliana* models^19^^,^^20^ (Figure 3; Data S4). The TSPOON TM scores for each species were high (*P. tricornutum* – 0.77, *Blastocystis* – 0.79), suggesting relatively strong structural conservation between species (Data S4A). The *Blastocystis* TSAUCER (0.52; Data S4B) and the *P. tricornutum* TPLATE (0.50; Data S4C) produced lower TM scores with the *A. thaliana* sequences, but these were nonetheless marginally higher than the values obtained for *D. discoideum*.

**Figure 4:**
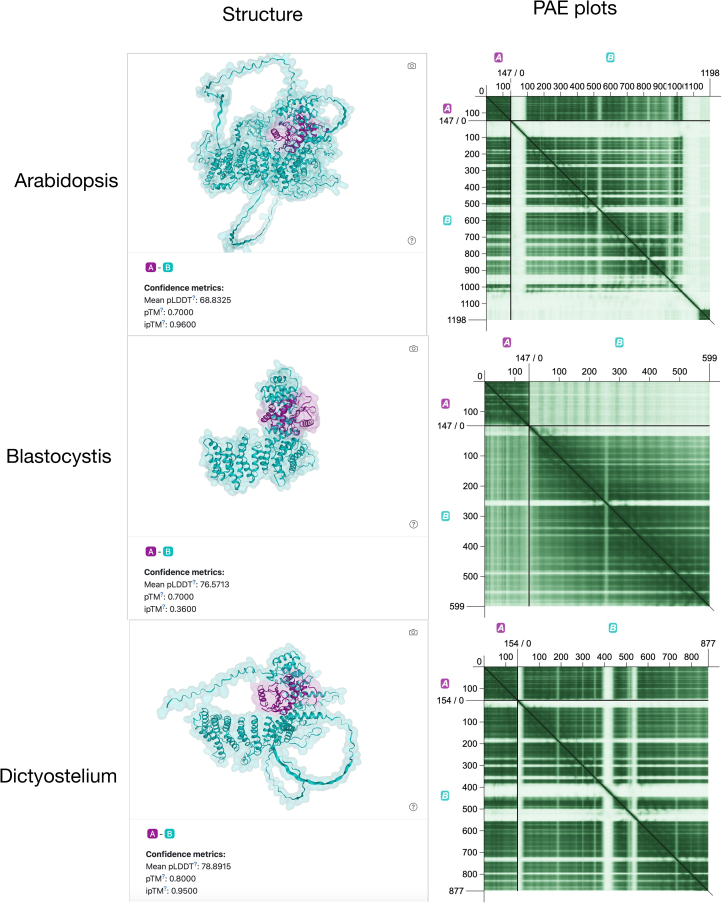
Subunit docking modeling of TSAUCER-TSPOON subunits For each of the pairs of TSAUCER and TSPOON from *A. thaliana*, *Blastocystis* sp., and *D. discoideum*, the structural models were predicted showing individual structures and potential interactions. Predicted structures are shown to the left, PAE plots shown to the right. pLDDT scores for each predicted structure are near, or above, the confidence threshold (>70), with the experimentally confirmed interacting pair from *A. thaliana* having the lowest score. The overall quaternary structure of the subunit pairs is relatively concordant.

Finally, since the structural interactions between subunit pairs within the TSET complex have been determined to a high resolution in the *A. thaliana* system, we wanted to know whether the subunit pairs of TSAUCER-TSPOON and TPLATE-TCUP were also modeled to bind with one another for the stramenopile orthologues. This would predict that the interactions known in *A. thaliana* are also occurring in the distantly related taxa (Data S5A). AlphaFold^36^ was used to model the structures of the TPLATE-TCUP pairs from *A. thaliana*, *Blastocystis*, and *P. tricornutum* (*D. discoideum* failed to compute) and the TSAUCER-TSPOON pairs from *A. thaliana*, *Blastocystis*, and *D. discoideum* (*P. tricornutum* not having a detected TSAUCER subunit). While the TPLATE-TCUP predictions for the stramenopiles were fairly low confidence (Data S5B), precluding drawing any strong conclusions, those for the TSAUCER-TSPOON pair (Figure 4) were within the confidence range, i.e., pLDDT>70. These suggest that the TSAUCER-TSPOON subunits could form a complex, one which at least visually resembles that modeled for *A. thaliana*.

Overall, the predicted structural similarity of the TSET subunits from the examined stramenopile representatives to characterized molecular cell biology models are suggestive of conserved functional roles.

### The μHD of TCUP, but not the SH3 domain of TSAUCER, are conserved features across eukaryotes

Previous studies reported that the TCUP and TSAUCER subunits exhibit a gain or loss of specific functional features during eukaryotic evolution. More precisely, the μHD domain of TCUP is reported to be absent in *D. discoideum* but present in *A. thaliana* where it mediates interactions with AtEH/Pan1, and the SH3 domain in TSAUCER is only found in Viridiplantae organisms (Chlorophyte and Streptophytes).^20^^,^^38^^,^^39^ Searches for conserved μHDs in our TCUP dataset reveals that the “μHD superfamily” domain (a subcategory of the AP muniscins-like MHD superfamily) is detected in nearly all protein sequences from glaucophytes, cryptophytes and haptophytes but in less than a quarter of those from chlorophytes and stramenopiles (Figure S3; Data S3). However, the “AP muniscins-like MHD superfamily” domain was detected across almost all sequences in every group analyzed. We interpret this seeming contradiction as a matter of detection sensitivity in the respective domain profiles, and overall conclude that the μHDs is present in the orthologues that we have detected, with varying levels of sequence conservation. In contrast, neither of these two domain profiles were found in the *D. discoideum* TCUP sequence, suggesting that the μHD feature of TCUP is conserved across eukaryotes but was subsequently lost in certain lineages. Conversely, the “SH3 superfamily” domain was exclusively detected in Chlorophytes, consistent with previous findings^39^ and extending them to a much larger set of taxa. Taken together, these results demonstrate that the TCUP and TSAUCER subunits of the TSET complex exhibit varying features across eukaryotes, while remaining components of the same conserved complex.

### Co-expression of *Phaeodactylumtricornutum* TSET and endomembrane trafficking genes

To further assess the possibility of functional homology of the stramenopile TSET components to the experimentally determined systems of *A. thaliana* and *D. discoideum*, we considered whether we could identify proteins that may interact with TSET in eukaryotic algae. Because of its use as a model organism for diatoms, an algal group of major ecological importance,^10^^,^^15^ we focused for this analysis on *P. tricornutum*. Specifically, to uncover genes with possibly linked biological functions, we searched for genes that are coregulated (expressed under the same conditions, with similar proportional abundances in each condition) as genes encoding TSET subunits, using a previously benchmarked protocol.^40^^,^^41^^,^^42^ Briefly, this involved utilizing a large *meta*-dataset of *P. tricornutum* gene expression (RNA-seq and microarray) data^40^^,^^41^ and calculating Spearman correlations (i.e., ranked transcript abundances) for gene expression between six annotated TSET subunits and all other genes across the version 3 annotation of the *P. tricornutum* genome. We ranked genes by their mean Spearman correlation value to *P. tricornutum* genes encoding four central TSET subunits (Phatr3_J43047 – TCUP, Phatr3_J54511 – TPLATE, Phatr3_J54718 – TSPOON, and Phatr3_J46356 – TTRAY) as evidence of positive coregulation.^42^ Two additional paralogues of TCUP (Phatr3_J43761) and TSPOON (Phatr3_J14536) were found to show limited coregulation to other TSET subunits, and excluded from the analysis. A summary of the subunits discussed below are provided in Figure S4, and complete data are available as a pivot table (Data S6).

Across the entire *P. tricornutum* genome, the most strongly positively coregulated genes (mean coregulation values >0.8 to the TSET core) include multiple endomembrane proteins: Phatr3_J43900, an ER membrane protein complex subunit 1; Phatr3_J10209, coatomer subunit gamma; Phatr3_J24186, exportin-1; Phatr3_J47327, exocyst complex component 7; and Phatr3_J49389, trafficking protein particle complex subunit 11. These may suggest possible roles for *P. tricornutum* TSET in exocytosis. Intriguingly, the most positively correlated protein with the TSET core is a *P. tricornutum* uncharacterized protein (Phatr3_J49389) with homology (at least within the C2-lipid binding domain) to another jötnarlog protein AGD12, a member of the ArfGAP_C2 protein subfamily,^43^ which acts within the Golgi body in plants and mediates gravitropism.^44^

**Figure 5:**
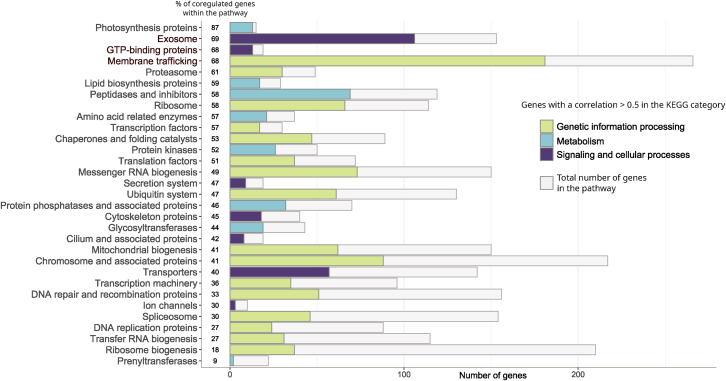
KEGG functions enriched in genes co-expressed with P. tricornutum TSET Gene co-regulated to four core TSET subunits (Phatr3_J43047 – TCUP, Phatr3_J54511 – TPLATE, Phatr3_J54718 – TSPOON and Phatr3_J46356 – TTRAY) was assessed by Spearman correlation of a *meta*-dataset of *P. tricornutum* RNA-seq and microarray data per.^42^ The numbers of coregulated genes (*r* > 0.5) in each category of the “Gene and proteins” classification of the KEGG Mapper Pathway are represented by colored bars, while gray bars represent the total number of genes in the corresponding category. Categories are sorted decreasingly from top to bottom based on their percentage of completeness (shown on the left of the bars). The “Enzymes” category (correlated = 579 genes, total = 1450 genes) and categories containing fewer than 5 genes are not considered. Raw supporting data are provided in Data S6.

Further KEGG analysis of genes associated with core TSET subunits (mean coregulation values >0.5, 2671 genes, 22% of total genes) confirms that the TSET complex may be associated with vesicular transport (Figure 5). Indeed, the “Exosome”, “GTP-binding proteins”, and “Membrane trafficking” categories ranked in the top 5 most complete functional categories (Figure 5), with respectively 69% (106 coregulated/153 total genes in the “Exosome” category), 68% (13/19 genes) and 68% (181/266 genes) of completeness. Among the “GTP-binding proteins”, several genes are referenced to be directly involved in intracellular protein transport or to be part of the Ras superfamily (Phatr3_J43251, ARF1; Phatr3_J8659, ARL1; Phatr3_J54420, SAR1A; Phatr3_J13157; and Phatr3_J45953). The “Photosynthesis proteins” category, including photosystem subunits, plastid electron transport chains and light-harvesting complex proteins, was ranked as the most complete category with 87% associated genes (13/15; Figure 5). Several other metabolic pathways also have a strong association with the core TSET such as the “Signal transduction” metabolic pathway (207 coregulated genes, 61% complete, 5^th^ rank) and the “Transport and catabolism” metabolic pathway (175 coregulated genes, 60% complete, 6^th^ rank), the latter containing genes involved in endocytosis (34 genes), lysosomal activity (20 genes) and autophagy processes.

Finally, based on previous reports from plants that TSET can dynamically associate with the actin cytoskeleton,^38^^,^^39^ we searched for actin, dynamin and tubulin-related proteins that showed a coregulation coefficient >0.7 with the four *P. tricornutum* TSET core subunits, alongside EF-hand motif proteins that might mediate these interactions. Among our dataset (Figure S4), we noted strong positive co-regulation to TSET genes of two probable *P. tricornutum* homologues of the AtEH1/Pan1 and AtEH2/Pan2 EF-hand proteins associated with TSET/actin interactions; (Phatr3_J30394, mean coregulation coefficient 0.790; Phatr3_J42442, mean coefficient 0.745).

Overall, these data suggest that in *P. tricornutum* the TSET complex is linked to key cellular processes such as vesicular trafficking, as demonstrated in *A. thaliana*^19^^,^^45^ and *D. discoideum*.^20^

### *Tara* Oceans data reveal the global presence and environmental significance of TSET

Finally, we wanted to understand whether TSET was playing a substantial role in real world settings. To assess this, we retrieved putative homologues of diatoms’ TSET proteins from *meta*-transcriptome data in the primary *Tara* Oceans expedition. This dataset covers 153 globally distributed sites, separated by depth (surface, and Deep Chlorophyll Maximum), and cell size (from between 0.8 and 2000 μm diameter), allowing quantitative assessment of gene expression in wild communities. Each sample within this dataset is also associated with environmental parameters (coordinate location, measured temperature, and measured or modeled nutrient concentrations including nitrogen, phosphorus, iron, and silicon),^10^ allowing preliminary assessments of the physiological functions of TSET in natural communities.

**Figure 6:**
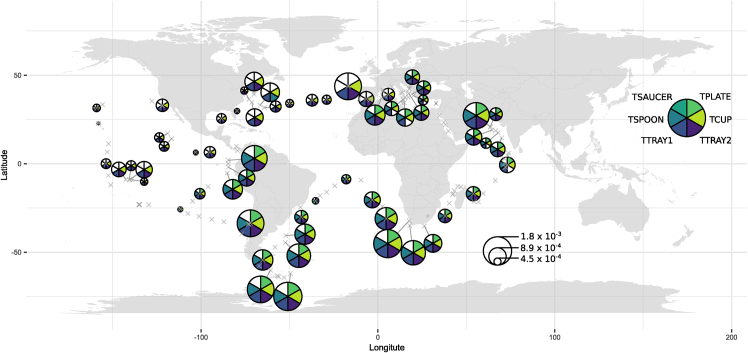
Map of Tara Oceans distributions of diatom TSET meta-transcripts Circles represent the mean between surface and DCM samples of the normalized total abundances of all putative TSET subunits transcripts found in each sampling station. The abundance of transcripts is expressed as the percentage of total transcript reads within the station, normalized by the total abundance of diatoms transcript reads within the same station. The presence or absence of the putative TSET subunits transcripts in a station is shown on the corresponding pie chart. Gray crosses indicate the location of all sampling stations. No occurrence of TSAUCER transcripts was detected.

Meta-transcripts encoding inferred diatom TSET subunits were detected across *Tara* Oceans data (Figure 6; raw data are provided in Data S7). Over the 67 stations where putative TSET transcripts were detected, all of them presented TCUP, TTRAY1, and TTRAY2, and 34 stations presented the additional TPLATE and TSPOON subunits (Figure 6). TSAUCER was never detected, a result consistent with those obtained in our comparative genomic approach (Figure 2). TSET components seem to be constitutively expressed in the wild diatom populations, with normalized abundances between 4.5 × 10^−4^ and 1.8 × 10^−3^ total relative abundance (Figure 6). Considering the relative expression of the homologous medium subunits of TSET (TCUP) and the related AP2 complex (AP2mu) within *Tara* Oceans dataset, we observed that AP2mu is typically more highly expressed than is TCUP (Figure S5). Nonetheless, the average TSET expression levels across all subunits, is comparable to that of enzymes with defined mutant phenotypes in diatoms, e.g., mitochondrial and plastid glycolysis,^46^ suggesting probable physiological significance. Moreover, meta-transcripts were identified in a wide diversity of diatom species (Figure S6), many of which were also detected in our comparative genomic approach (Figure 2, Data S2), demonstrating consistency across our analyses.

**Figure 7:**
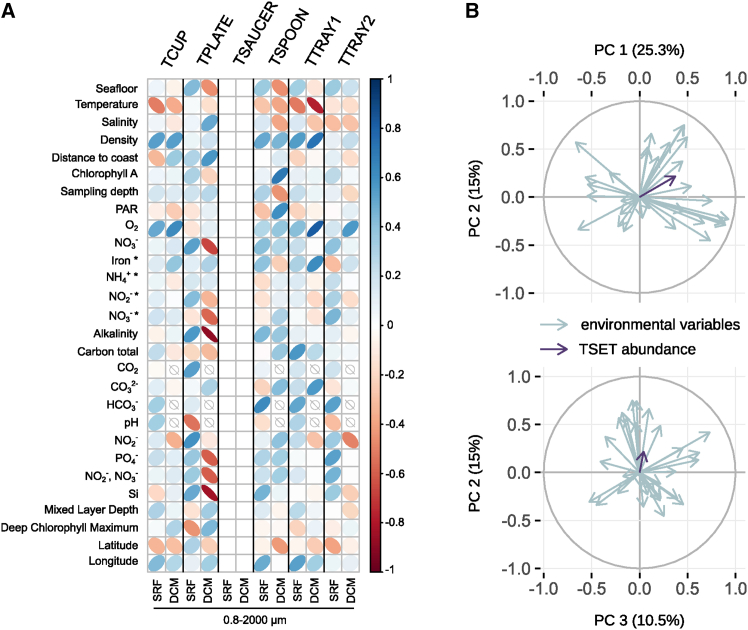
Correlations between the relative abundances of diatom TSET meta-transcripts and *Tara* Oceans environmental variables (A) Heatmap of the Spearman correlation coefficients between relative transcript abundances and environmental parameters. Normalized relative abundances for each sampling stations were calculated for samples corresponding to the 0.8 to 2000 μm size fraction, collected at the surface (SRF) or at the Deep Chlorophyll Maximum (DCM). Shapes and colors of the ellipses correspond to the correlation coefficient value, empty signs show that no value is available for the corresponding environmental parameter. The “TSAUCER” column is blank as no transcripts were detected. (B) Principal component analysis of *Tara* environmental conditions and TSET relative abundances. “TSET abundance” corresponds to the normalized total abundances of all putative TSET subunits meta-transcripts found in each sampling station, depth and size fraction. Environmental parameters used for the PCA are listed in A, with the minimal and maximal O_2_ depths added. PC1 is positively associated with nitrogen and phosphorus nutrient concentrations, PC2 is positively associated with carbon nutrient and pH, PC3 is associated with depth parameters (salinity, density, photosynthetically active radiations).

Visually, TSET expression appears to be most abundantly expressed in stations from high latitude southern and coastal stations, and more variably highly expressed at northern stations, with lower normalized abundance in the tropical open ocean (Figure 6). A global correlation analysis specifically projects negative correlations between meta-transcript abundance and temperature, and positive correlations to O_2_ content and seawater density for multiple TSET subunits (Figure 7A). That said, a principal component analysis (PCA) did not identify any one single environmental factor as having a predominant correlation with normalized TSET meta-transcript abundances (Figure 7B). Overall, our data are consistent with diatom TSET being pervasively and perhaps basally expressed in the world ocean.

## Discussion

In this study, we harness systems-level and environmental data to characterize a taxonomically widely distributed but poorly understood membrane complex, TSET. This was done in five evolutionarily heterogeneous protist and algal lineages that play substantial roles in marine ecosystems, and are of agricultural, industrial, and medical significance. Notably, beyond the initial description of the TSET complex, the distribution of the complex had not been assessed in a systematic way and instead was couched in analyses focused on the presence or absence of the genes encoding the complex in a given organismal genome. Our study comprehensively, and with an emphasis on broad taxonomic sampling, explores in which of these algal groups TSET is found and, secondly, what functions it may perform.

Prior to this work, the TSET complex had been reported sporadically in the algal groups in question, with some reports of complete complexes in members of the green algae,^30^ and in the stramenopile *Blastocystis*,^31^ but with quite sporadic distribution in stramenopiles in general.^20^ Here, we project much more widespread occurrence of TSET in algae than previously identified (i.e., from small numbers of genomes). TSPOON and TPLATE are most frequently detected, even in the absence of other subunits (cf., haptophyte TSPOON), and it is possible that they have functions as monomers (similarly to opisthokont TCUP, which acts without the other core TSET subunits in the clathrin accessory FEI complex^25^). TTRAY and TCUP, in particular, have more sporadic distributions. These proteins have limited structural conservation (AlphaFold TM scores <0.2) even between the two model species (*D. discoideum* and *A. thaliana*) in which they were first characterized, and perhaps they are under-detected in our libraries due to sequence divergence. Finally, TSAUCER is unusual, as we rarely detect it in algal groups with otherwise complete TSET complexes (e.g., stramenopiles including diatoms), although we could slightly expand our detection using taxon-specific search queries (e.g., additional dictyochophyte homologues found using the *D. speculum* sequence).

With respect to the question of TSET’s cellular role in these important algal groups, the evidence suggests that the complex is likely to serve homologous functional roles embedded in the distinct biology of the organisms in question. We show that TSPOON, TPLATE and TSAUCER show moderate to strong reciprocal AlphaFold conservation (TM score >0.5) between *A. thaliana* and *D. discoideum.* While TSET plays different roles for plants and amebae, its involvement in the basic cellular processes of endomembrane trafficking is conserved in both organisms. The TSPOON and TPLATE subunits of the two stramenopile models studied (*P. tricornutum* and *B. hominis*), and the TSAUCER subunit of *B. hominis*, have TM scores with *A. thaliana* that meet or exceed those of *D. discoideum*, suggesting their structural roles in the TSET complex are conserved. This is true of the individual subunit structural models and when the subunit pairs of TSAUCER and TSPOON are modeled together. Gene co-regulation analysis in the model diatom *P. tricornutum* suggest that four TSET subunits (TCUP, TTRAY1, TSPOON, and TPLATE) are co-expressed with genes involved in endomembrane trafficking, including links with the cytoskeleton and Pan1/2 (eps15 homologues). The Pan1 and Pan 2 proteins are the result of a recent duplication in dicotyledons of the eps15 protein,^23^ which is otherwise found broadly in eukaryotes. Though Pan1/2 are part of the TPC complex and eps15 is part of the FEI complex in animals,^25^ eps15 also appears to play roles in non-CME pathways at the PM^26^ and is found in some taxa that do not possess any TSET subunits, such as *Giardia intestinalis* and *Trypanosoma brucei*.^20^^,^^47^ Therefore, we hesitate to conclude that the eps15 homologues in *P. tricornutum* are part of a TSET complex or whether the eps15 is a core member of TSET ancestrally and generally present in eukaryotes. This exciting possibility requires more extensive genomic sampling across eukaryotic diversity and experimental strategies, e.g., crosslinking and pull-down, and proximity proteomics, both of which are now established for *Phaeodactylum* *tricornutum* membrane proteins.^48^^,^^49^ Nonetheless our structural modeling and gene co-regulation analyses suggest that the role of the TSET complex in endomembrane processes is conserved.

Finally, from *Tara* Oceans data, we detect meta-genes for a complete diatom TSET (barring TSAUCER) in many ocean provinces. The relative abundance of TSET meta-transcripts does not seem to be linked to a specific environmental parameter, suggesting that its expression may either be constant or under complex regulation inside the diatom cell. Nonetheless, our data are consistent with TSET playing a role in endomembrane trafficking in important organisms for health, agriculture, and the environment.

More broadly, our data reinforce two major themes. First, the importance of using phylogenetically diverse protist and algal references for understanding the significance of broadly conserved cellular processes. Second, that jötnarlog genes such as TSET may be widespread across the eukaryote tree, and can be expressed in wild populations. The cellular processes associated with classical opisthokont models (e.g., yeast and animals) may not always be applicable to other taxa, creating paradoxical situations where genes that are found widely across eukaryotes can still have unresolved functions or where important genes for eukaryotic cell biology are not integrated into generally applied cell biological frameworks. Development of model organisms from plants (*A. thaliana*), and a few key parasites (*Toxoplasma*, *Trypanosoma*, and to a lesser extent *Giardia, Plasmodium, Trichomonas*, and *Entamoeba*) have been instrumental in bringing this bias to light. However, because plants have unique multicellular biology, and because parasites are often highly reduced, diverged, and/or specialized, with cryptic homology to opisthokonts, the broader relevance of their cell function has been historically underappreciated. Where they present clear concurrence with opisthokonts, they are integrated into general cell biological models, and where there is discordance, they are often unfairly discounted. Complementing our findings here, focused on a single jötnarlog protein complex in many taxa, a recent study^50^ highlighted the high prevalence, and thus relevance, of 16 jötnarlogs in the genomes of several disparately related free-living heterotrophic groups. This included diplonemid flagellates, which are highly diverse and abundant marine protists. Here, we advocate for an even broader perspective, integrating algae, protists, and phylogenetically diverse eukaryotic organisms into fundamental cell biology research.^15^ This could provide several benefits to the field, allowing us to understand cellular bases of a wide range of behavioral and trophic strategies, and providing a better proxy to understand molecular coevolution. Moreover, *Tara* Oceans and other sampling campaigns provide huge amounts of quantitative expression data alongside repeatedly measured environmental variables. These ecologically important microscopic organisms, and their uncultured relatives, combined with the wealth of underexplored proteins (jötnarlogs) in membrane trafficking may hold particular promise in revealing fundamental processes in evolutionary cell biology.

### Limitations of the study

Our dataset was designed to maximize taxonomic inclusion and likely incorporates false negatives due to sequencing depth and low expression of TSET subunits in the conditions under which the organisms were cultured. We have attempted to mitigate this with multiple search strategies. Nonetheless, the conclusions regarding the overall paucity of identified TSET subunits in red algae, cryptophytes, and haptophytes with some exceptions (e.g., haptophyte TSPOON) and the inference that suggests they play a minor role in these taxa is based on lineage-wide and not individual homology detection results. The highly sporadic distribution of stramenopile TSAUCER, and the evidence that genes encoding other diatom TSET subunits are co-regulated and expressed in the wild, may suggest that TSAUCER is actually present in these groups, but escaping detection by our informatic methods. A more convoluted scenario that TSAUCER has been convergently and repeatedly replaced by an analogous protein in stramenopiles is also possible, though we believe less likely.

Second, the conclusions that we make regarding structural homology between the TSET orthologues, and the dependent inferences about whether TSET complexes may have conserved functions between stramenopiles and plants or amoebae, are dependent on the reliability of the structural predictions from the modeling methods. While we did see low TM scores for many of the orthologue-orthologue comparisons that we made, we also note that those pairs comprise individual proteins with low pLDDT scores as well (Data S4D–S4F), thus indicating that the data themselves are unreliable. By contrast, we did observe pLDDT scores for many of the subunits above 70, thus falling in the confidence range for well-predicted protein structures (Data S4G).^51^ Consequently, we used two criteria to restrict our conclusions to the most reliable data. First, we started the analysis by comparing the pairs of subunits where functional homology had been experimentally established (i.e., *D. discoideum* and *A. thaliana),* and setting the TM scores for those as a benchmark for what we should expect for the kind of functional homology that we are considering. Second, we did set a lower limit based on the general usage of TM scores, which is that scores over 0.5 are indicative of structural conservation. Notably, in those cases where we draw conclusions about proposed homology, all of the individual subunits also had mean pLDDT between 65 and 95, with the lowest score being actually from the *A. thaliana* TSAUCER model (65.31). For the subunit interactions, we applied a similar confidence criteria and only drew conclusions if the pLDDT score for the three subunit pair models were ∼70. We note that AlphaFold itself does not model TSET individual subunits or pairs with high reliability, with even the *A. thaliana* subunits, for which structures have been determined experimentally, often falling below the confidence ranges. Hopefully, this will improve as more experimental data are collected and integrated into structural modeling tools such as AlphaFold.

Finally, though we have chosen the conservative definition “involvement in endomembrane pathways”, our inferences concerning the cellular roles of diatom TSET are speculative. This relates to the indirect relationship between gene co-expression and function, and the differences between algal cell biology and that of plants and amebae in which TSET was first characterized. The potential functions of TSET in eukaryotic algae will be best investigated by molecular approaches. First, structural homology could be determined by complementation of plant or other TSET mutant lines with algal proteins to test for restoration of non-mutant phenotypes. Second, reverse genetics in transformable diatoms such as *P. tricornutum* could provide insights into TSET localization (e.g., via GFP-labeling across the cell cycle), or function (e.g., via growth or omic analysis of CRISPR knockout lines). Finally, IP-pulldowns or sub-cellular proteomics could determine whether a diatom TSAUCER orthologue or analogue exists, and identify the TSET interactome, as was recently done for *A. thaliana*.^52^

## Resource availability

### Lead contact

Further information and requests for resources and reagents should be directed to and will be fulfilled by the lead contact, Joel B. Dacks (dacks@ualberta.ca).

### Material availability

This study did not generate new unique reagents.

### Data and code availability

• This paper used publicly available datasets, available at https://osf.io/ykxes/.
• This paper does not report original code.
• Any additional information required to reanalyze the data reported in this paper is available from the lead contact upon request.

## Acknowledgments

We wish to thank members of the Dacks and Dorrell labs for helpful suggestions and support, particularly Dr. Kristína Záhonová. Work in the Dorrell lab is supported by an 10.13039/501100001665ANR JCJC (“PanArctica”, ANR-21-CE02-0014, awarded 2021–2022) and an 10.13039/100010663ERC Starting Grant (“ChloroMosaic”, 101039760, awarded 2023–2027). Work in the Dacks lab is supported by grants from the 10.13039/501100000038Natural Sciences and Engineering Research Council of Canada (RES0043758 and RES0046091).

## Author contributions

J.B.D. designed the study. M.P.-R. led the comparative genomics with help from M.S., K.M.F., and R.L. M.P.-R. performed *Tara* Oceans analyses, and M.S. performed AlphaFold structural comparisons. R.G.D. provided reference sequence datasets and performed gene co-regulation analyses. M.P.-R., R.G.D., and J.B.D. wrote the paper, with input from all other coauthors. All coauthors read and approved the manuscript draft prior to submission.

## Declaration of interests

The authors have no competing interests to declare.

## STAR★Methods

### Key resources table

**Table:** 

REAGENT or RESOURCE	SOURCE	IDENTIFIER
**Software**		

AMEBAE	33	https://github.com/laelbarlow/amoebae
Seaview	58	RRID:SCR_015059
ClustalO	59	RRID:SCR_001591
Gblocks	60	RRID:SCR_015945
AlphaFold2	36	RRID:SCR_025454
PAEViewer	50	https://doi.org/10.1093/nar/gkad350
Bugaco converter	NA	http://sequenceconversion.bugaco.com/converter/biology/sequences/fasta-2line_to_stockholm.php
HMMER 3.3.2	NA	RRID:SCR_005305; http://hmmer.org
R 4.2.1	NA	RRID:SCR_001905
BLAST	NA	RRID:SCR_004870; https://blast.ncbi.nlm.nih.gov/Blast.cgi

### Method details

#### Reference library construction

A composite reference library of 266 stramenopile, 47 haptophyte, 281 green algal (including 9 glaucophyte), 42 red algal and 34 cryptophyte genomes and transcriptomes (Data S1) was compiled from previous studies.^54^^,^^55^ This consisted of publicly accessible genomes, accessed from JGI PhycoCosm (07/22), decontaminated MMETSP and OneKp transcriptome libraries for each group, plus eighteen further chrysophyte and seven further diatom (both stramenopile) transcriptomes independently sequenced in other studies. Libraries were hierarchically organised according to the results of recent multigene phylogenies of each lineage^56^^,^^57^^,^^58^ and were divided into groups of broadly equivalent rank to orders. For example, stramenopiles were divided into Oomycetes, Labyrinthulomycetes, Pelagophytes, Pinguiophytes, Dictyochophytes, Phaeophytes and Xanthophytes (PX), Raphidophytes, Diatoms, Eustigmatophytes, Synurophytes and Chrysophytes (SC), Bolidophytes, Bicosoecids, and *Blastocystis*; and haptophytes were divided into Pavlovophytes, Phaeocystales, Prymnesiales, Isochrysidales, and Coccolithales-Syracosphaerales-Zygodiscales (CSZ) (Data S2). The full taxonomically sorted library is available for user exploration at https://osf.io/ykxes/.

#### Homologue detection

Searches for putative TSET subunit homologues (Data S2) were performed using the AMEBAE workflow. Protein sequences of confirmed TSET components (Data S3) were collected from Hirst et al.^20^ and aligned on Seaview using ClustalO (default parameters).^59^^,^^60^ These alignments were used as queries in AMEBAE, a semi-automatic reverse search tool, using the buit-in hmmsearch 3.3.1 algorithm. Several reference genomes were used for the AMEBAE searches: *Arabidopsis thaliana* (GCA_000001735), *Dictyostelium discoideum* (GCF_000004695), *Phaeodactylum tricornutum* (GCA_000150955), *Blastocystis* sp. *(*previously *hominis)* (GCA_000151665), and *Emiliana huxleyi* (GCA_000372725). The same queries, references and Ref_seqs_1_manual_predictions.csv files were used for independent searches in stramenopile, haptophyte, red algal, green algal, and cryptophyte datasets. For stramenopiles and haptophytes, searches were performed using a dataset composed of both NCBI reference genomes (69 and 6, respectively, accessed 10/10/2023) and the custom reference dataset supplied above (Data S1). For each AMOEBAE search, all four AP1 subunits sequences from the fungi *Rhizophagus irregularis* were used as positive controls, this complex being present in every eukaryote examined thus far.^33^ Organisms in which fewer than two AP1 subunits were detected were considered as not complete enough and thus not retained for further analyses.

Stramenopile and haptophyte TSET putative homologues were aligned with the query sequences and their AP/COP paralogues (outgroup) in Seaview using ClustalO (default parameters).^59^^,^^60^ Alignments were trimmed in Seaview using Gblocks (options for a less stringent selection),^59^^,^^61^ and phylogenetic trees were computed in Seaview using PhyML with default parameters (LG model, aLRT branch support). Putative homologue sequences that did not group with the reference sequences were considered as false positives and removed. Successive rounds of selection were performed to obtain a topology with clear monophyly of each TSET subunit. Complete homologue lists, alignments, and curated tree topologies are provided in Data S3.

#### Conserved domain analysis

Putative TCUP and TSAUCER sequences from Data S3 were searched on the web-based CD-search tool (accessed on 04/12/2024, https://www.ncbi.nlm.nih.gov/Structure/bwrpsb/bwrpsb.cgi),^62^ with default parameters (search mode automatic; CDD -- 62456 PSSMs database; e-value threshold = 0.01; composition-corrected scoring = TRUE; Maximum number of hits = 500). The results were downloaded (Data mode = Full) and normalized to the initial number of sequences used for each group. Sequences from *Arabidopsis thaliana* and *Dictyostelium discoideum* were used as positive and negative controls, respectively.

#### Structural modelling

AlphaFold was performed using AlphaFold2 on google Colab using defaults settings and computational systems^36^^,^^63^ (Data S4). The predicted structure were viewed along with the predicted aligned error plot on PAEVIewer.^51^ TM scores were calculated on the online portal.^37^ A similar approach was used to model interactions between TPLATE and TCUP (Data S5).

#### Phaeodactylum co-regulation analysis

To identify genes co-expressed with TSET subunits in the *P. tricornutum* genome, a meta-dataset of previously published gene expression data was used, per previous studies^42^^,^^54^ (Data S6). Broadly, this consisted of two normalised meta-studies of RNAseq (PhaeoNet)^40^ and microarray data (DiatomPortal),^41^ with relative transcript abundances or fold-changes (respectively) transformed into ranked values, where 100 corresponded to the most highly expressed gene in the dataset, and 0 the lowest. Genes not evaluated in each study were marked as N/A to avoid biasing the correlation values obtained. Pearson correlations of ranked gene expression abundances (i.e., Spearman correlations) were calculated for all genes in the version 3 annotation of the *P. tricornutum* genome^64^ and six genes encoding TSET subunits: Phatr3_J43047 (TCUP1), Phatr3_J43761 (TCUP2), Phatr3_J54511 (TPLATE), Phatr3_J54718 (TSPOON1), Phatr3_J14536 (TSPOON2), and Phatr3_J46356 (TTRAY). An average correlation value was calculated for all six subunits, and for a “core” set of TCUP1, TPLATE, TSPOON1 and TTRAY, as TCUP2 and TSPOON2 were found to have limited transcriptional coregulation with other TSET subunits.

*Phaeodactylum* genes were annotated following previous studies for identified epigenetic marks (histone acetylation and methylation, DNA methylation, and polycomb group presence), predicted function (GO, PFAM, KEGG), and predicted localisation of the encoded protein (ASAFind, HECTAR, MitoFates, WolfPSort).^40^ Complete tabulated coregulation coefficients and predicted protein annotations are provided in Data S6. The KEGG Mapper Pathway (accessed 26/10/2023) was fed with all K numbers listed in the Data S6 in order to determine the cellular processes correlated with the expression of the “core” TSET genes, with a correlation coefficient threshold >0.5. The pathway composition obtained with all the gene from the version 3 of the *P. tricornutum* genome was used as a reference to calculate the completeness of each pathway after the correlation filter, i.e., the percentage of genes from a specific pathway which are coregulated with the “core” TSET genes.

#### Tara Oceans analysis

For each TSET subunit, diatom homologues were aligned in Seaview using ClustalO.^59^^,^^60^ Alignments were converted in stockholm format using the online Bugaco converter (http://sequenceconversion.bugaco.com/converter/biology/sequences/fasta-2line_to_stockholm.php), and then converted into an HMM file using the hmmbuild function of HMMER 3.3.2.

Putative homologous transcripts were retrieved from the *Tara* Oceans metatranscriptomic dataset thanks to the online Ocean Gene Atlas tool (https://tara-oceans.mio.osupytheas.fr/ocean-gene-atlas/), using the following options: MATOUv1+T dataset, 1 × 10^−10^ expect threshold, abundances as percent of total reads, and the custom HMM file as the query. The putative homologue abundances were downloaded, as well as the associated environmental parameters (Data S7). Only sequences annotated as diatoms (Bacillariophyta) were used for analyses. We believe this preliminary taxonomic annotation is sufficient to classify diatom TSET sequences due to the absence of evident horizontal transfers of its subunits within cultured species. Transcript abundances were normalised for each station, depth and size fraction using the total transcript abundance for diatoms in the corresponding sample, in order to prevent biases due to the ecological preferences of these organisms.^65^ Analyses were conducted using R version 4.2.1 and the ggplot2, corrplot, factoextra, FactoMineR, VIM and missMDA packages.

For TSAUCER, no diatom homologue was available. A hmmer search was performed using the TSAUCER reference query sequences alignment (see homologue detection), with no success.

### Quantification and statistical analysis

#### Domain retention analysis

Calculation of the percentage of sequences in which conserved domains were detected. No statistical test was performed, and details are found in the legend and on the figure of Figure S3, and in the "conserved domain analysis" part of the Method section.

#### Gene Co-regulation

Calculation of KEGG functions enriched in co-expressed genes in *P. tricornutum* as the percentage of coregulated genes in the pathway relatively to the total number of genes in this pathway. No statistical test was performed, details can be found in the legend and on the figure of Figure 4.

#### Tara Oceans analysis

Mean abundance of TSET transcripts at each station. Details can be found in the legend of Figure 5.

Spearman correlation coefficient between relative transcript abundances and environmental variables found on Figure 6A. No statistical test was performed.

PCA conducted on environmental conditions and transcript abundances. No statistical test was performed, details can be found in the legend and on the figure of Figure 6B.

Calculation of the ratio between TCUP and AP2mu metatranscripts in each station. No statistical test was performed, and details are found in the legend.

## Supporting citation

The following reference appears in the supplemental information: Keeling et al.^53^

## Supplemental information

Document S1. Figures S1–S6 and Data S4 and S5

Data S1. Composition of the multispecies reference library and taxonomic divisions used for TSET comparative genomic analysis, related to Figures 1 and 2 Data are available at https://osf.io/ykxes/.

Data S2. TSET composition of the species used for this study as inferred by AMOEBAE search, related to Figures 1 and 2 Table of AMOEBAE search results for TSET subunits, sorted by taxonomy. Only genomes/transcriptomes with two or more AP1 subunits detected are presented.

Data S3. Datafiles used in informatic analyses, related to Figures 1 and 2 List of TSET homologues, sequences of query TSET proteins, alignments and curated tree topologies, and Ref_seqs_1_manual_predictions.csv used for the AMOEBAE searches.

Data S6. Coregulation coefficients of six recovered TSET subunit genes Coregulation coefficients of six recovered TSET subunit genes -Phatr3_J43047 (TCUP1), Phatr3_J43761 (TCUP2), Phatr3_J54511 (TPLATE), Phatr3_J54718 (TSPOON1), Phatr3_J14536 (TSPOON2), and Phatr3_J46356 (TTRAY) – and all other genes in the Phaeodactylum tricornutum genome version 3 annotation, assessed by Spearman correlation analysis of merged RNA-seq (PhaeoNet) and microarray (DiatomPortal) data, related to Figure 5. Sheet 1 of Data S6 (“Phat3 genome") provides an overview of the entire genome and its associated transcriptional dynamics. Columns A to C provide each gene, its ID and full-length peptide sequence. The first six rows correspond to inferred TSET homologues. Columns D to M provide information concerning the expression trends associated with each gene: its occurrence in DiatomPortal and PhaeoNet coexpressed gene modules; and its calculated Spearman coregulation coefficient to each inferred TSET gene, all six TSET genes (“TSET average”) or the average coregulation coefficient calculated purely for TCUP1, TPLATE, TSPOON1, and TTRAY (“Core average”). Columns N to R provide information concerning the inferred localization of the protein encoded by each model, considering the consensus of ASAFind (realized with SignalP 3.0), HECTAR, MitoFates (with threshold value 0.35) and WolfPSort (consensus animal, plant and animal annotations). Columns S to Z provide information concerning the inferred function of the encoded protein, considering KEGG annotation (based on BLASTKOALA, GhostKOALA, KofamKOALA), PFAM and predicted biological process. Columns AA to AE provide associated genomic contexts, considering chromosomal localization, epigenetic marks (histone methylation and acetylation, and DNA methylation), and presence of inferred alternative splice-forms from mapped RNA-seq data. Sheet 2 of Data S6 (“Core enrichments”) provides a chi-squared pivot table of PhaeoNet RNA-seq WGCNA merged modules, consensus localization predictions, and consensus KEGG annotations enriched in genes with moderate (r > 0.5) or strong (r > 0.7) positive coregulation to the four core TSET subunits (TCUP1, TPLATE, TSPOON1, and TTRAY), as defined above. P-values are signed so that occurrences that are less frequently observed than expected are negative, and occurrences that are more frequently observed than expected are positive.

Data S7. Meta-transcript Tara Oceans data used to perform TSET analysis in diatoms, related to Figures 6 and 7 Tabulated *Tara* Oceans TSET homologues and relative abundances in different stations, depths and size fractions.
