# Supplementary material for: Prevalence and environmental abundance of the TSET complex in cosmopolitan algal groups
Source: iScience. 2025 May 15;28(6):112679. doi: 10.1016/j.isci.2025.112679 (PMC12167817; doi:10.1016/j.isci.2025.112679)
Supplement: Document S1. Figures S1–S6 and Data S4 and S5 [file mmc1.pdf]

iScience, Volume *28*

Supplemental information

Prevalence and environmental abundance of the TSET complex in cosmopolitan algal groups

Mathias Penot-Raquin, Mandeep Sivia, Kelly M. Fafoumi, Raegan Larson, Richard G. Dorrell, and Joel B. Dacks

iScience, Volume ■■

Supplemental information

Prevalence and environmental abundance of the TSET complex in cosmopolitan algal groups

Mathias Penot-Raquin, Mandeep Sivia, Kelly M. Fafoumi, Raegan Larson, Richard G. Dorrell, and Joel B. Dacks

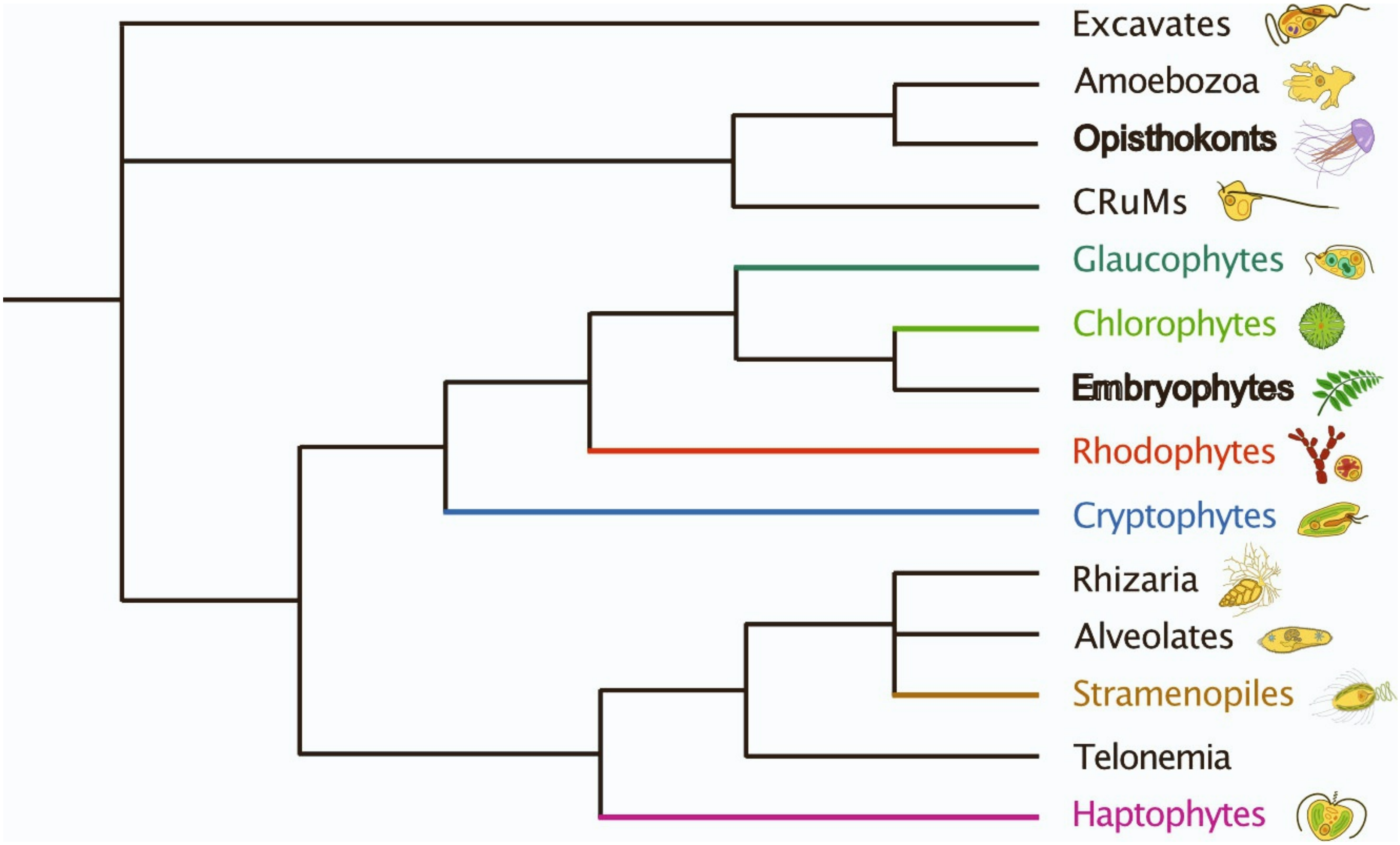

Figure S1: Schematic tree of eukaryotic life, highlighting the major algal groups studied in this paper

Taxa containing classical model organisms in cell biology are in bold, coloured taxa are the ones on which this study focuses [1]. Branch lengths are arbitrary, and exact tree topology is still under debate. Thumbnail images are from [53]. Related to Figures 1 and 2.

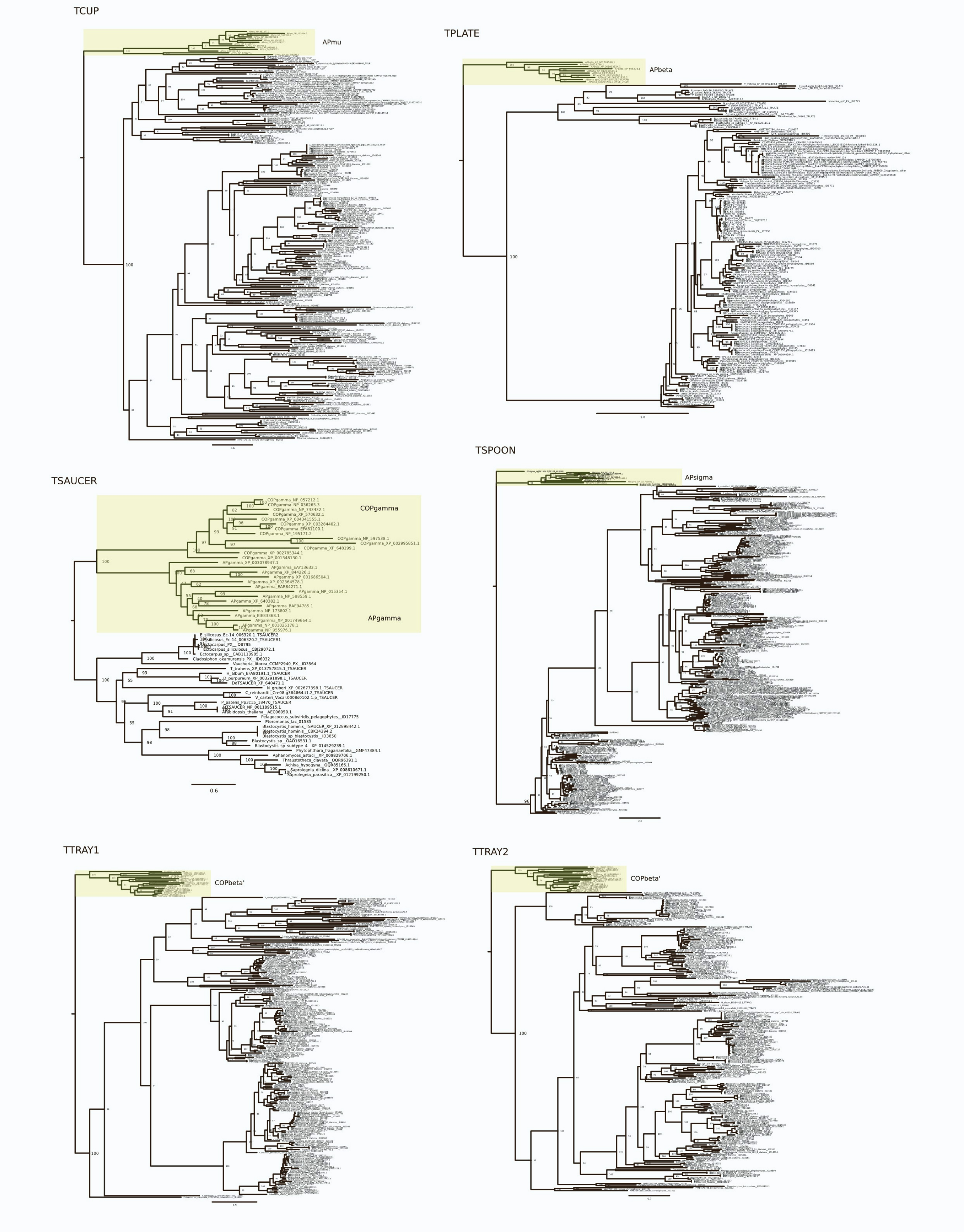

Figure S2: Phylogenies of TSET subunits demonstrate a vertical inheritance of this complex through eukaryotic evolution.

Consensus trees of stramenopile and haptophyte TSET subunits, Related to Figures 1 and 2. Phylogenies were computed with IQ-TREE2 v2.2.6. The VT+R7 model (best model assessed by ModelFinder for TCUP) was used for each tree. Branch supports are ultrafast bootstrap approximation (1000 replicates). Outgroups correspond to homologous AP or COP subunits. at least visually consistent, suggestive of conserved pairwise subunit interactions.

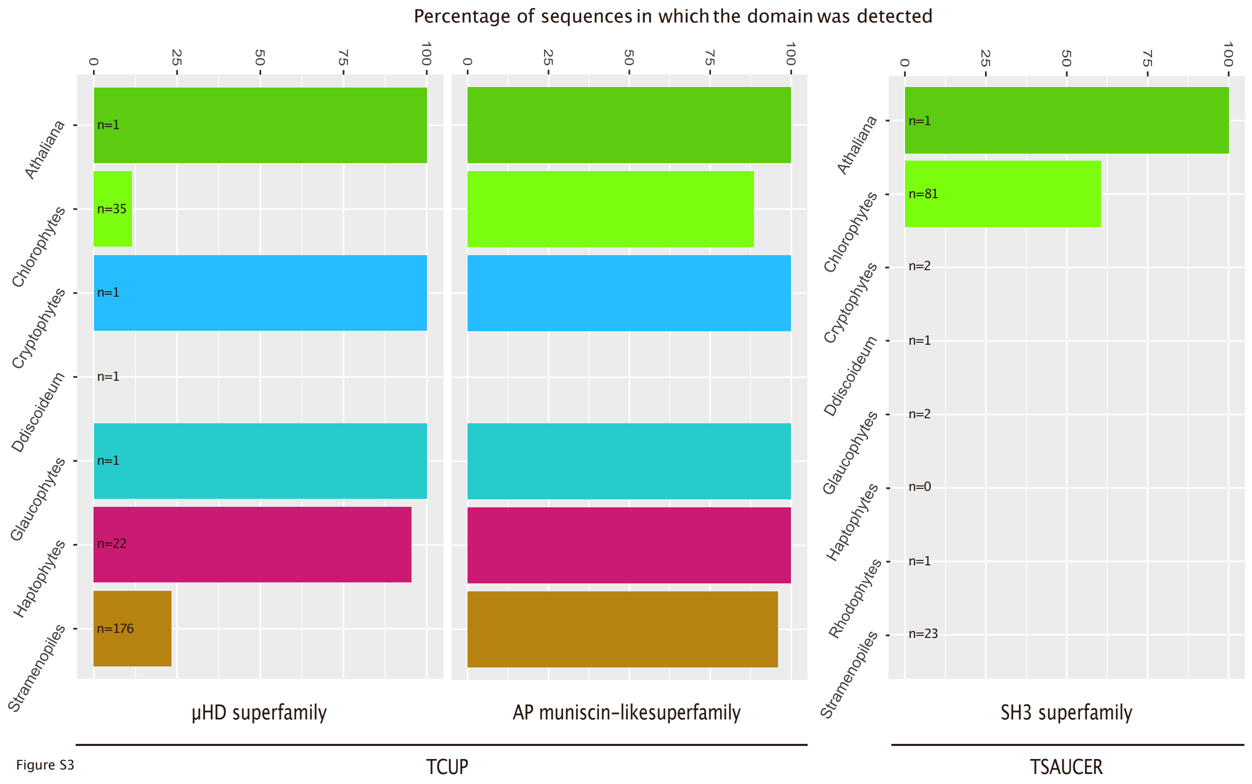

Figure S3. The TCUP µHD domain is a conserved feature, lost in Dictyostelium, while TSAUCER SH3 domain is specific to the Viridiplantae.

Percentage of sequences in which the conserved domain (indicated at the bottom of each panel) was detected using the Conserved Domain search. TCUP and TSAUCER sequences from *A. thaliana* and *D. discoideum* were used as positive and negative controls, respectively. The number of input sequences for each subunit and group is indicated at the base of the bars. Note that no TCUP sequences are available for Rhodophytes, and no TSAUCER sequences are available for Haptophytes (Figure 1, Data S3). Related to Figure 1.

Genename	Identity	PhaeoNet	Average	TCUP1	TPLATE	TSPOON1	TTRAY	KOFAM	PFAM
Phatr3_J43047	TCUP1	skyblue	0.777	1.000	0.873	0.502	0.735		MuniscinC–terminalmuhomologydomain
Phatr3_J43761	TCUP2	darkgrey	-0.010	0.100	0.029	-0.149	-0.021		Adaptorcomplexesmediumsubunitfamily
Phatr3_J54511	TPLATE	grey	0.803	0.873	1.000	0.681	0.658	Coatomersubunitbeta	AdaptinNterminalregion
Phatr3_J54718	TSPOON1	orangered4	0.659	0.502	0.681	1.000	0.452	AP–4complexsubunitsigma–1	Clathrinadaptorcomplexsmallchain
Phatr3_J14536	TSPOON2	orange	0.231	0.170	0.351	0.489	-0.086	AP–3complexsubunitsigma	Clathrinadaptorcomplexsmallchain
Phatr3_J46356	TTRAY	darkgrey	0.711	0.735	0.658	0.452	1.000		
Phatr3_J49389	endomembraneprotein	brown	0.858	0.911	0.905	0.796	0.822	traffickingproteinparticlecomplexsubunit11	Gryzun,putativetraffickingthroughGolgi
Phatr3_J47327	endomembraneprotein	darkmagenta	0.833	0.854	0.881	0.859	0.739	exocystcomplexcomponent7	exocystcomplexsubunit
Phatr3_J43900	endomembraneprotein	orange	0.826	0.892	0.910	0.786	0.714	ERmembraneproteincomplexsubunit1	Proteinofunknownfunction(DUF162)
Phatr3_J47270	endomembraneprotein	brown	0.825	0.829	0.910	0.853	0.709		Sec23/Sec24
Phatr3_J24186	endomembraneprotein	salmon	0.825	0.789	0.900	0.857	0.753	exportin–1	Importin–beta;Exportin1–like
Phatr3_J10209	endomembraneprotein	darkgrey	0.823	0.826	0.971	0.839	0.654	coatomersubunitgamma	AdaptinNterminalregion
Phatr3_J45854	endomembraneprotein	brown	0.801	0.862	0.898	0.616	0.829	nuclearporecomplexprotein	Nuclearprotein96
Phatr3_J16891	endomembraneprotein	orange	0.801	0.830	0.867	0.790	0.716	AP–4complexsubunitepsilon–1	AdaptinNterminalregion;
Phatr3_J43251	endomembraneprotein	darkgrey	0.741	0.729	0.918	0.778	0.537	ARF1	ADP–ribosylationfactorfamily
Phatr3_J13157	endomembraneprotein	green	0.701	0.747	0.839	0.719	0.498		ADP–ribosylationfactorfamily
Phatr3_J54420	endomembraneprotein	orangered4	0.668	0.655	0.880	0.710	0.428	SAR1A	ADP–ribosylationfactorfamily
Phatr3_J45953	endomembraneprotein	cyan	0.654	0.632	0.652	0.695	0.640	mitochondrialRhoGTPase1	domainofDAPkinase
Phatr3_J8659	endomembraneprotein	darkgrey	0.597	0.635	0.767	0.595	0.392	ARL1	ADP–ribosylationfactorfamily
Phatr3_J31156	Actin/cytoskeletonprotein	brown	0.841	0.894	0.944	0.790	0.734		Dyneinheavychain
Phatr3_J47936	Actin/cytoskeletonprotein	brown	0.814	0.887	0.909	0.740	0.721	cytoskeleton–associatedprotein5	
Phatr3_J29136	Actin/cytoskeletonprotein	green	0.812	0.798	0.914	0.893	0.646		Actin
Phatr3_J29317	Actin/cytoskeletonprotein	cyan	0.805	0.792	0.884	0.895	0.650	cullin1	Cullinfamily
Phatr3_J15613	Actin/cytoskeletonprotein	darkgrey	0.743	0.749	0.928	0.785	0.512	cofilin	Cofilin/tropomyosin
Phatr3_J30898	Actin/cytoskeletonprotein	cyan	0.742	0.682	0.847	0.854	0.584	vacuolarprotein–sorting–associatedprotein4	Microtubuleinteractingandtransportdomain
Phatr3_J35252	Actin/cytoskeletonprotein	green	0.709	0.772	0.851	0.623	0.588	cappingprotein(actinfilament)	F–actincappingproteinalphasubunit
Phatr3_J44089	Actin/cytoskeletonprotein	darkgrey	0.705	0.668	0.866	0.792	0.496		Actin
Phatr3_J44183	Actin/cytoskeletonprotein	brown	0.705	0.693	0.623	0.724	0.780	SH3domain–containingYSC84–likeprotein1	Las17–bindingproteinactinregulator
Phatr3_J30394	AtEH1/Pan1homologue	darkgrey	0.775	0.790	0.880	0.836	0.594	EHdomain–containingprotein1	Cytoskeletal–regulatorycomplexEFhand
Phatr3_J42442	AtEH1/Pan1homologue	darkgrey	0.744	0.745	0.863	0.668	0.702		Cytoskeletal–regulatorycomplexEFhand
Phatr3_J1745	EFhandprotein	cyan	0.798	0.781	0.871	0.847	0.693	serine/threonine–proteinphosphatase2A	EF–handdomainpair
Phatr3_J31322	EFhandprotein	darkgrey	0.795	0.859	0.882	0.797	0.644		domainpair
Phatr3_J15479	EFhandprotein	brown	0.765	0.776	0.851	0.801	0.630	calcium–dependentproteinkinase	Proteinkinasedomain;EF–handdomain
Phatr3_J49771	EFhandprotein	tan	0.759	0.793	0.794	0.728	0.721		EFhand–like
Phatr3_J23794	EFhandprotein	darkgrey	0.749	0.722	0.891	0.862	0.522	calmodulin	EF–handdomainpair
Phatr3_J25067	EFhandprotein	brown	0.749	0.738	0.827	0.759	0.671	calcium–dependentproteinkinase	Proteinkinasedomain;EFhand
Phatr3_J14762	EFhandprotein	cyan	0.747	0.769	0.877	0.768	0.576		dehydrogenase
Phatr3_J1875	EFhandprotein	cyan	0.746	0.705	0.842	0.812	0.625	calcium–dependentproteinkinase	Proteinkinasedomain;EFhand
Phatr3_J6974	EFhandprotein	orangered4	0.735	0.667	0.809	0.891	0.574		EF–handdomain;EF–handdomainpair
Phatr3_J44174	EFhandprotein	cyan	0.734	0.761	0.866	0.739	0.568		EF–handdomainpair
Phatr3_J17199	EFhandprotein	magenta	0.729	0.827	0.875	0.625	0.590		domainpair
Phatr3_J40998	EFhandprotein	red	0.703	0.632	0.819	0.861	0.498	calcium–bindingproteinCML	EF–handdomainpair

Figure S4. *P. tricornutum* genes which strongly correlate with TSET are involved in endomembrane trafficking related functions.

Full outputs are provided in Data S6. The PhaeoNet merged module, Kofam and PFAM annotations of each gene are adapted from [40]. Related to Figure 5.

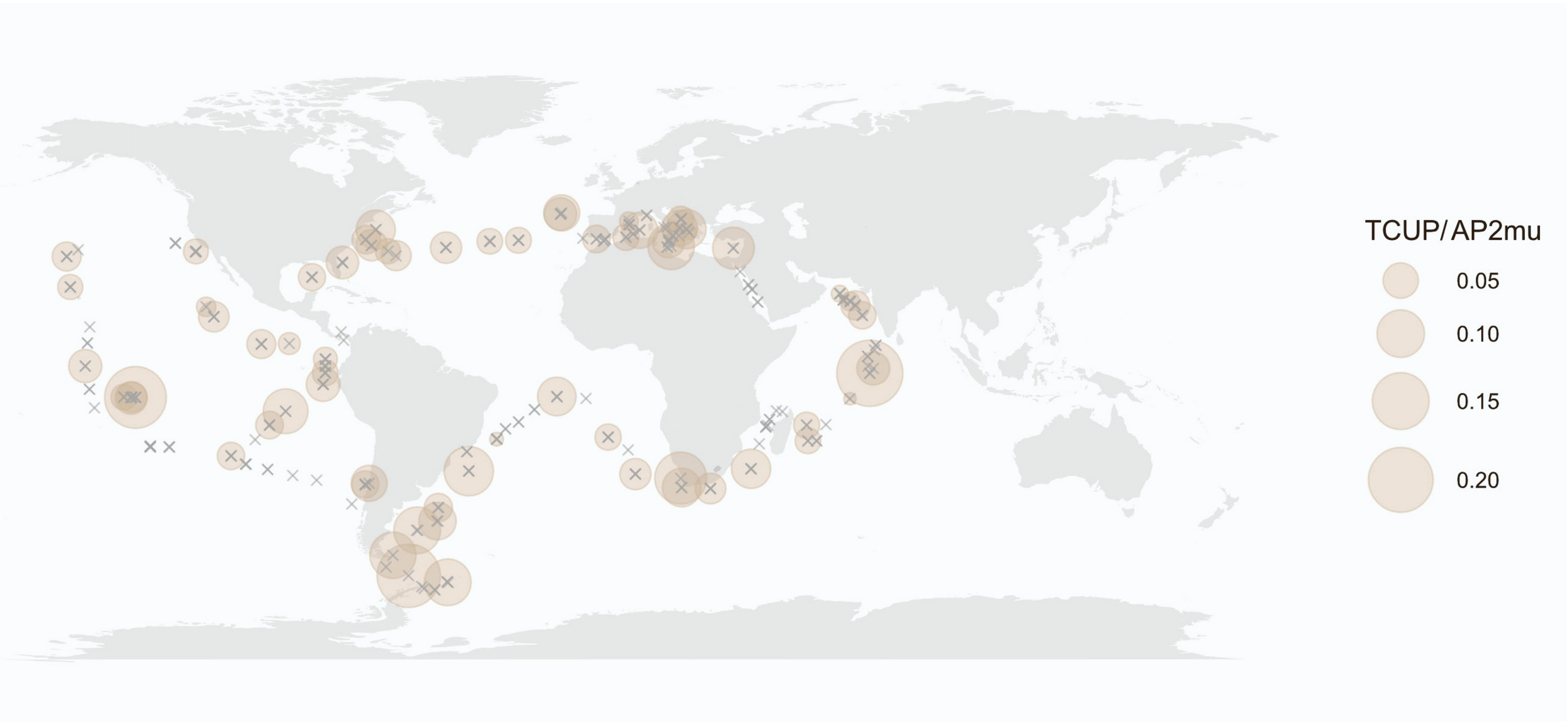

Figure S5. Lower relative abundances of TCUP than its homolog AP2mu are observed in diatom meta-transcriptome data.

Circles represent the ratio between the mean total abundances of all putative TCUP and APmu subunits transcripts found in each sampling station. The abundance of transcripts is expressed as the percentage of total transcript reads within the station. Grey crosses indicate the location of all sampling stations. Related to Figure 6.

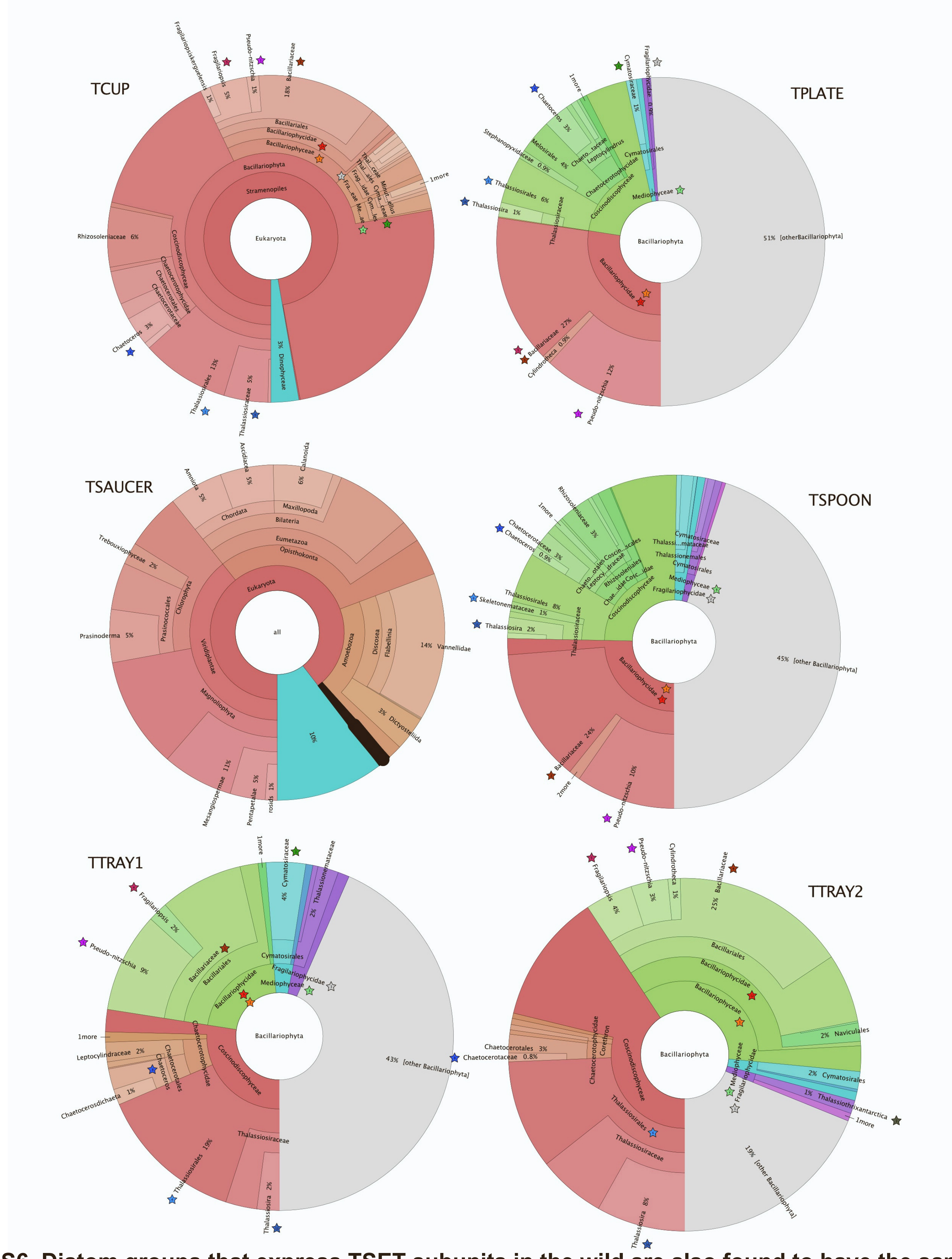

Figure S6. Diatom groups that express TSET subunits in the wild are also found to have the same TSET subunits in our comparative genomics survey.

Diversity and concordance of diatom species containing putative TSET subunits in *Tara* Oceans and comparative genomic studies. Krona plots generated via the Ocean Gene Atlas for each TSET subunits illustrate the diversity of diatoms from which sequences were detected. Stars indicate the number of corresponding subunits identified in our comparative genomic analysis (Figure 2, Data S2), shown at the highest accessible taxonomic level. Related to Figure 2.

Data S4: TM Align comparisons of AlphaFold predicted structures from all TSET subunits.

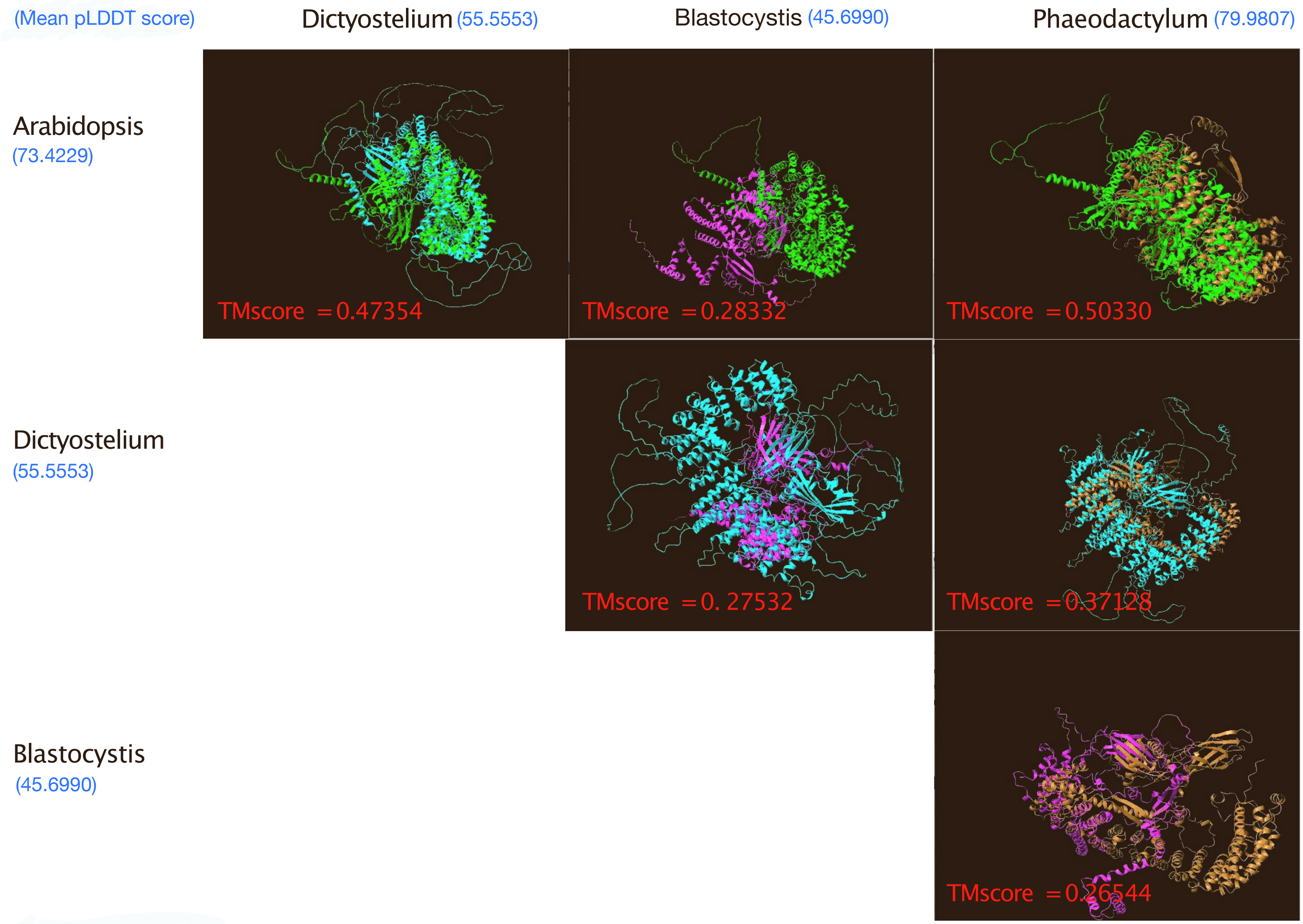

Data S4A: TM Align comparisons of AlphaFold predicted structures from all TSET subunits

All subunits from the TSET complex were predicted and aligned between all pairwise combinations of orthologues from *A. thaliana*, *D. discoideum*, *Blastocystis* sp., and *P. tricornutum*. TM scores are presented as normalised to the structure from the organism in the row. This gives two support values for each structural relationship, providing additional points for understanding structural similarity. Both TM scores of a relationship surpassing the 0.5 score threshold would indicate strong structural similarity. pLDDT scores for each predicted structure are provided, with a score >70 indicating confidence in the prediction, and >90 indicating high confidence. A: TSPOON. B. TSAUCER. C: TPLATE. D: TRAY1. E: TCUP. F: TTRAY2. G: PAE plots for all predicted subunits. Structural models shown in Figure 3 are highlighted in yellow, with the PAE graphs showing relatively reliable predictions, consistent with their TM scores near or above 0.5. Related to Figure 3.

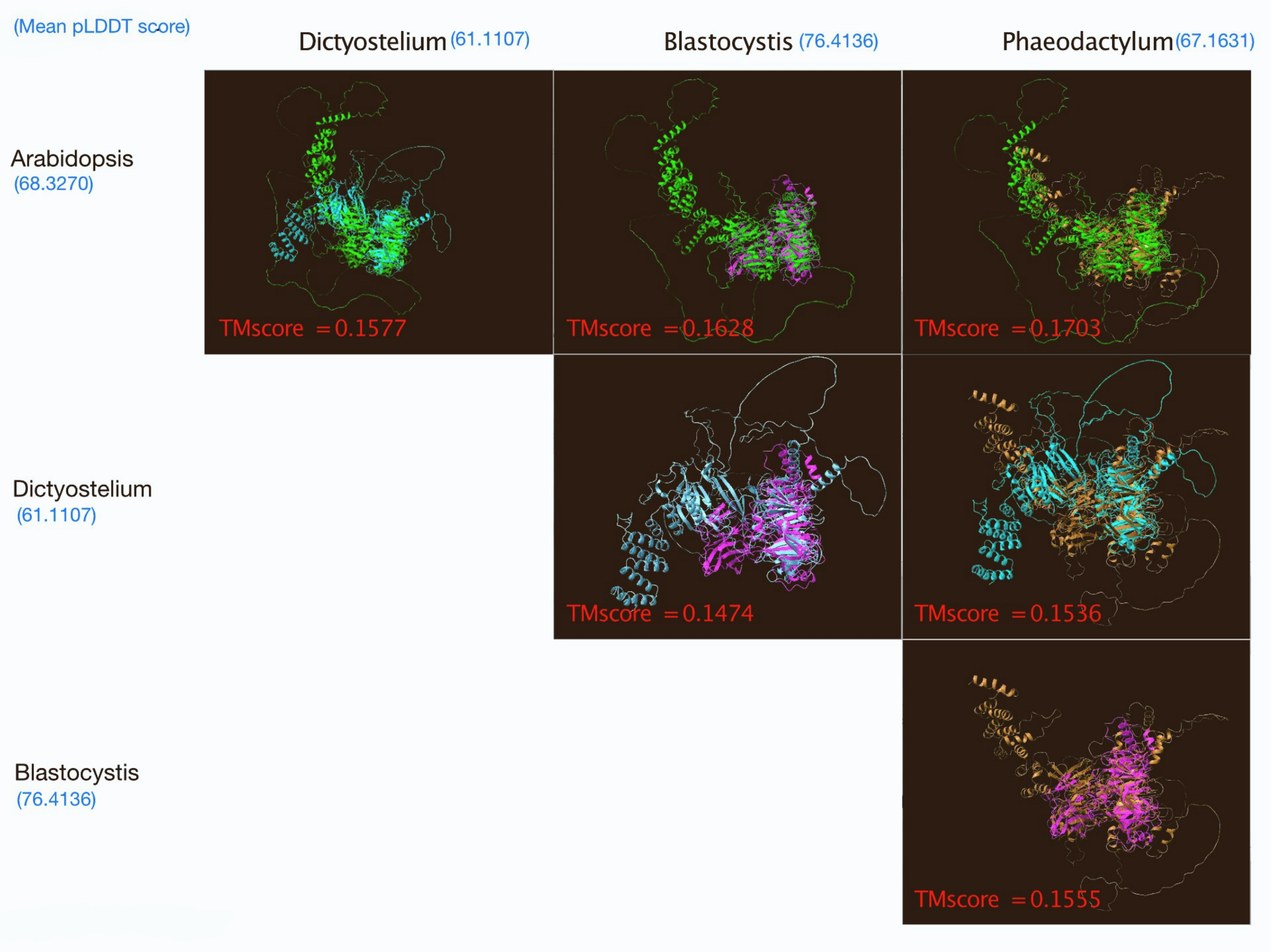

Data S4B: TM Align comparisons of AlphaFold predicted structures from all TSET subunits

All subunits from the TSET complex were predicted and aligned between all pairwise combinations of orthologues from *A. thaliana*, *D. discoideum*, *Blastocystis* sp., and *P. tricornutum*. TM scores are presented as normalised to the structure from the organism in the row. This gives two support values for each structural relationship, providing additional points for understanding structural similarity. Both TM scores of a relationship surpassing the 0.5 score threshold would indicate strong structural similarity. pLDDT scores for each predicted structure are provided, with a score >70 indicating confidence in the prediction, and >90 indicating high confidence. A: TSPOON. B. TSAUCER. C: TPLATE. D: TRAY1. E: TCUP. F: TTRAY2. G: PAE plots for all predicted subunits. Structural models shown in Figure 3 are highlighted in yellow, with the PAE graphs showing relatively reliable predictions, consistent with their TM scores near or above 0.5. Related to Figure 3.

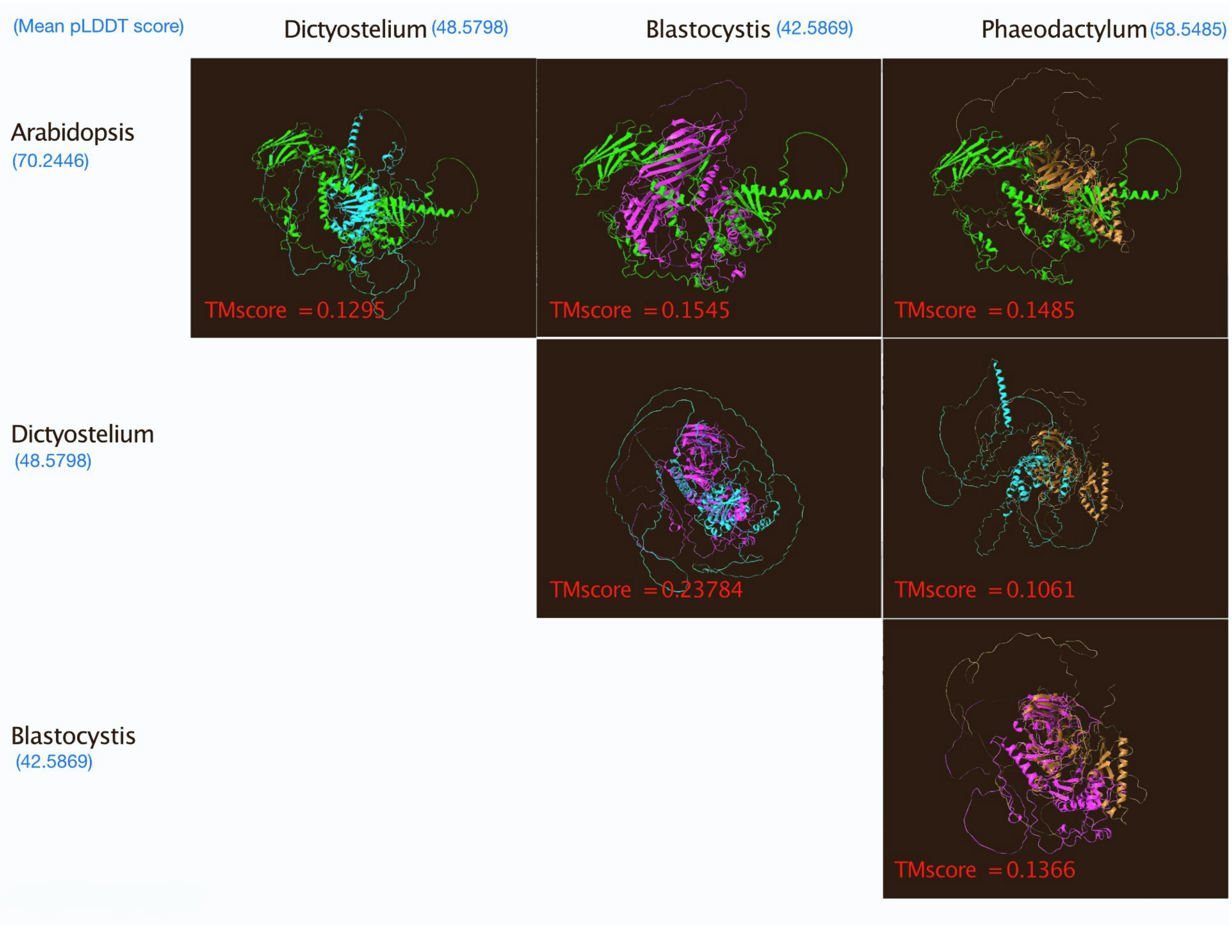

Data S4C: TM Align comparisons of AlphaFold predicted structures from all TSET subunits

All subunits from the TSET complex were predicted and aligned between all pairwise combinations of orthologues from *A. thaliana*, *D. discoideum*, *Blastocystis* sp., and *P. tricornutum*. TM scores are presented as normalised to the structure from the organism in the row. This gives two support values for each structural relationship, providing additional points for understanding structural similarity. Both TM scores of a relationship surpassing the 0.5 score threshold would indicate strong structural similarity. pLDDT scores for each predicted structure are provided, with a score >70 indicating confidence in the prediction, and >90 indicating high confidence. A: TSPOON. B. TSAUCER. C: TPLATE. D: TRAY1. E: TCUP. F: TTRAY2. G: PAE plots for all predicted subunits. Structural models shown in Figure 3 are highlighted in yellow, with the PAE graphs showing relatively reliable predictions, consistent with their TM scores near or above 0.5. Related to Figure 3.

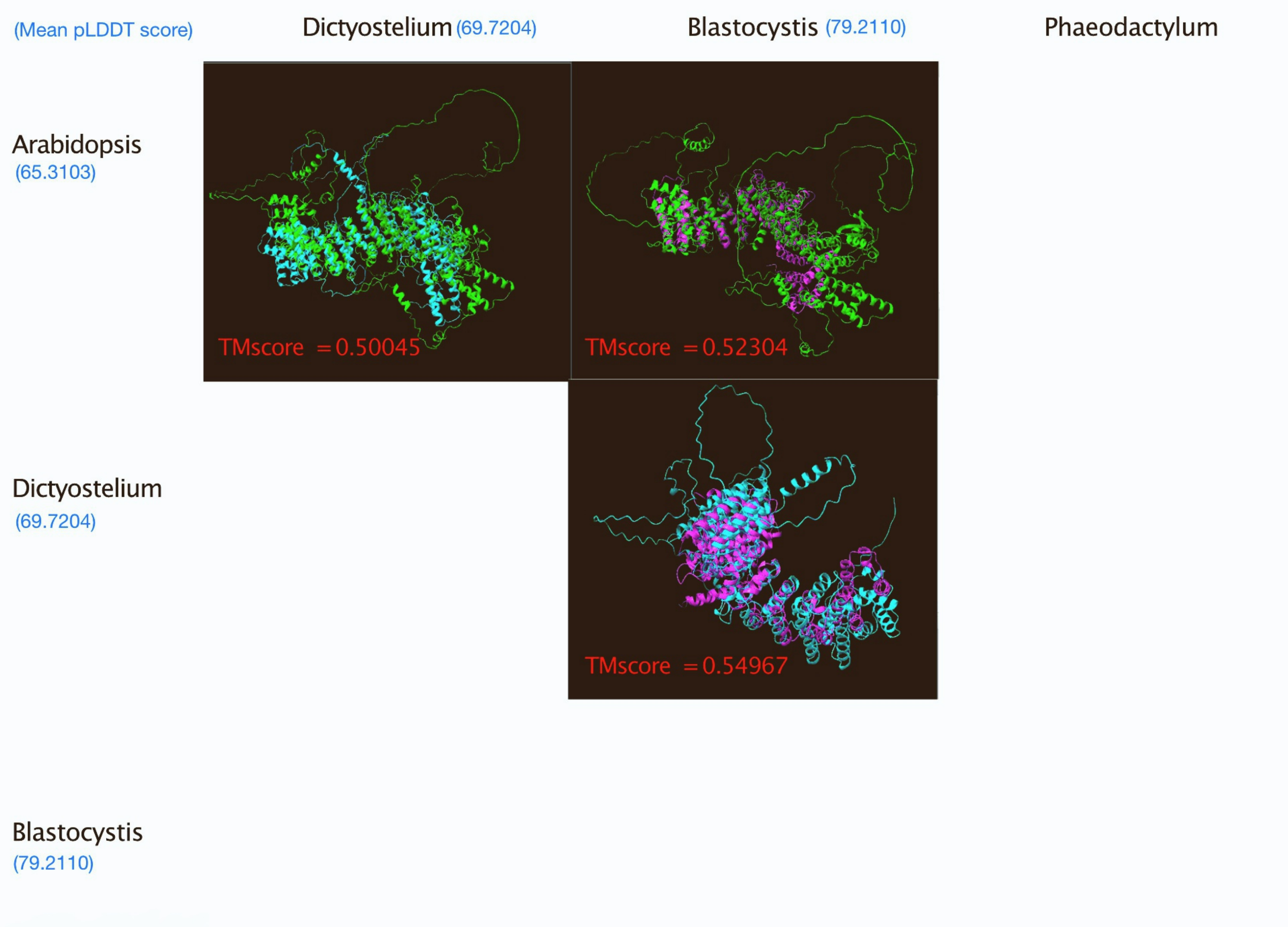

Data S4D: TM Align comparisons of AlphaFold predicted structures from all TSET subunits

All subunits from the TSET complex were predicted and aligned between all pairwise combinations of orthologues from *A. thaliana*, *D. discoideum*, *Blastocystis* sp., and *P. tricornutum*. TM scores are presented as normalised to the structure from the organism in the row. This gives two support values for each structural relationship, providing additional points for understanding structural similarity. Both TM scores of a relationship surpassing the 0.5 score threshold would indicate strong structural similarity. pLDDT scores for each predicted structure are provided, with a score >70 indicating confidence in the prediction, and >90 indicating high confidence. A: TSPOON. B. TSAUCER. C: TPLATE. D: TRAY1. E: TCUP. F: TTRAY2. G: PAE plots for all predicted subunits. Structural models shown in Figure 3 are highlighted in yellow, with the PAE graphs showing relatively reliable predictions, consistent with their TM scores near or above 0.5. Related to Figure 3.

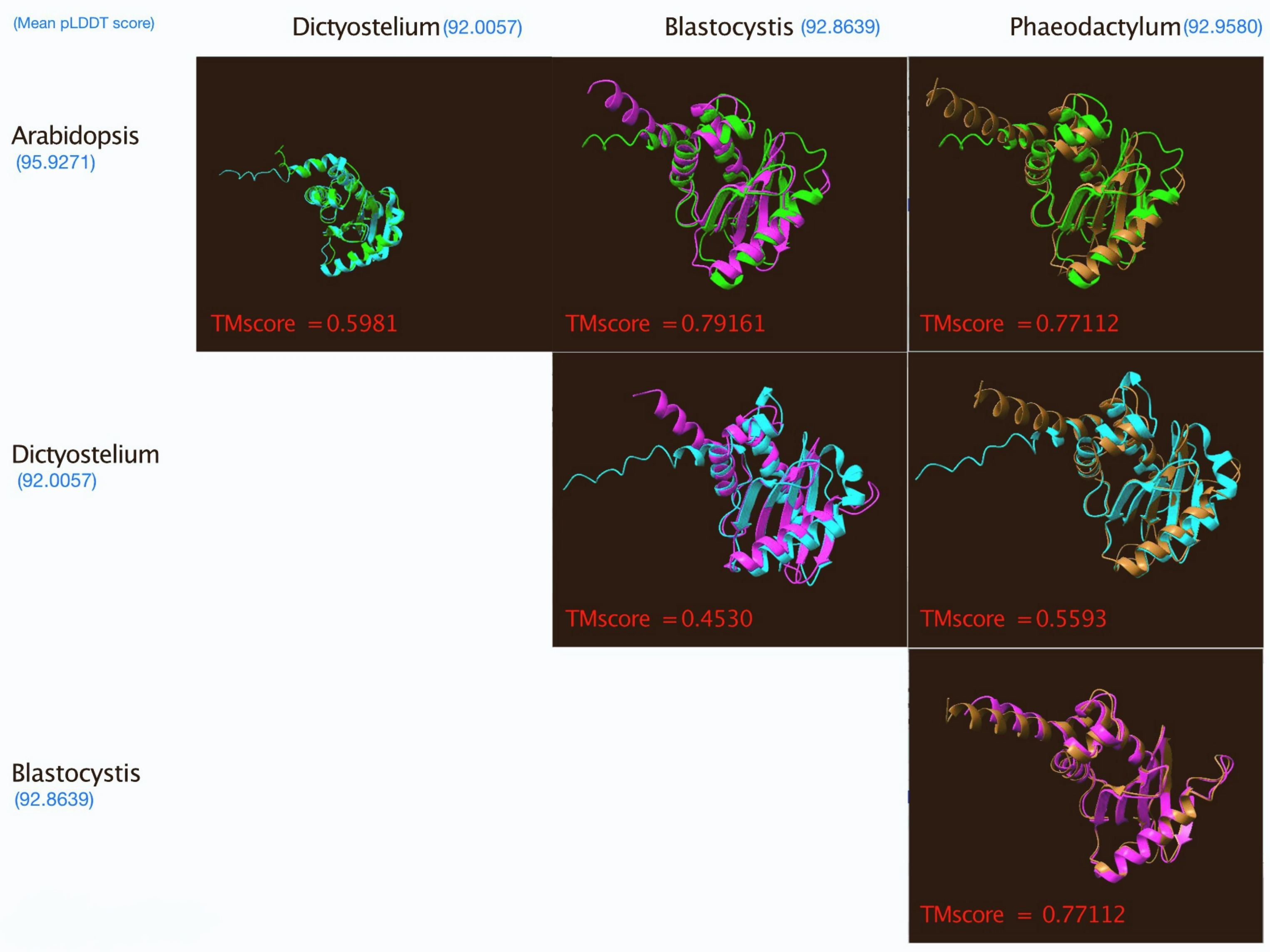

Data S4E: TM Align comparisons of AlphaFold predicted structures from all TSET subunits
All subunits from the TSET complex were predicted and aligned between all pairwise combinations of orthologues from *A. thaliana*, *D. discoideum*, *Blastocystis* sp., and *P. tricornutum*. TM scores are presented as normalised to the structure from the organism in the row. This gives two support values for each structural relationship, providing additional points for understanding structural similarity. Both TM scores of a relationship surpassing the 0.5 score threshold would indicate strong structural similarity. pLDDT scores for each predicted structure are provided, with a score >70 indicating confidence in the prediction, and >90 indicating high confidence. A: TSPOON. B. TSAUCER. C: TPLATE. D: TRAY1. E: TCUP. F: TTRAY2. G: PAE plots for all predicted subunits. Structural models shown in Figure 3 are highlighted in yellow, with the PAE graphs showing relatively reliable predictions, consistent with their TM scores near or above 0.5. Related to Figure 3.

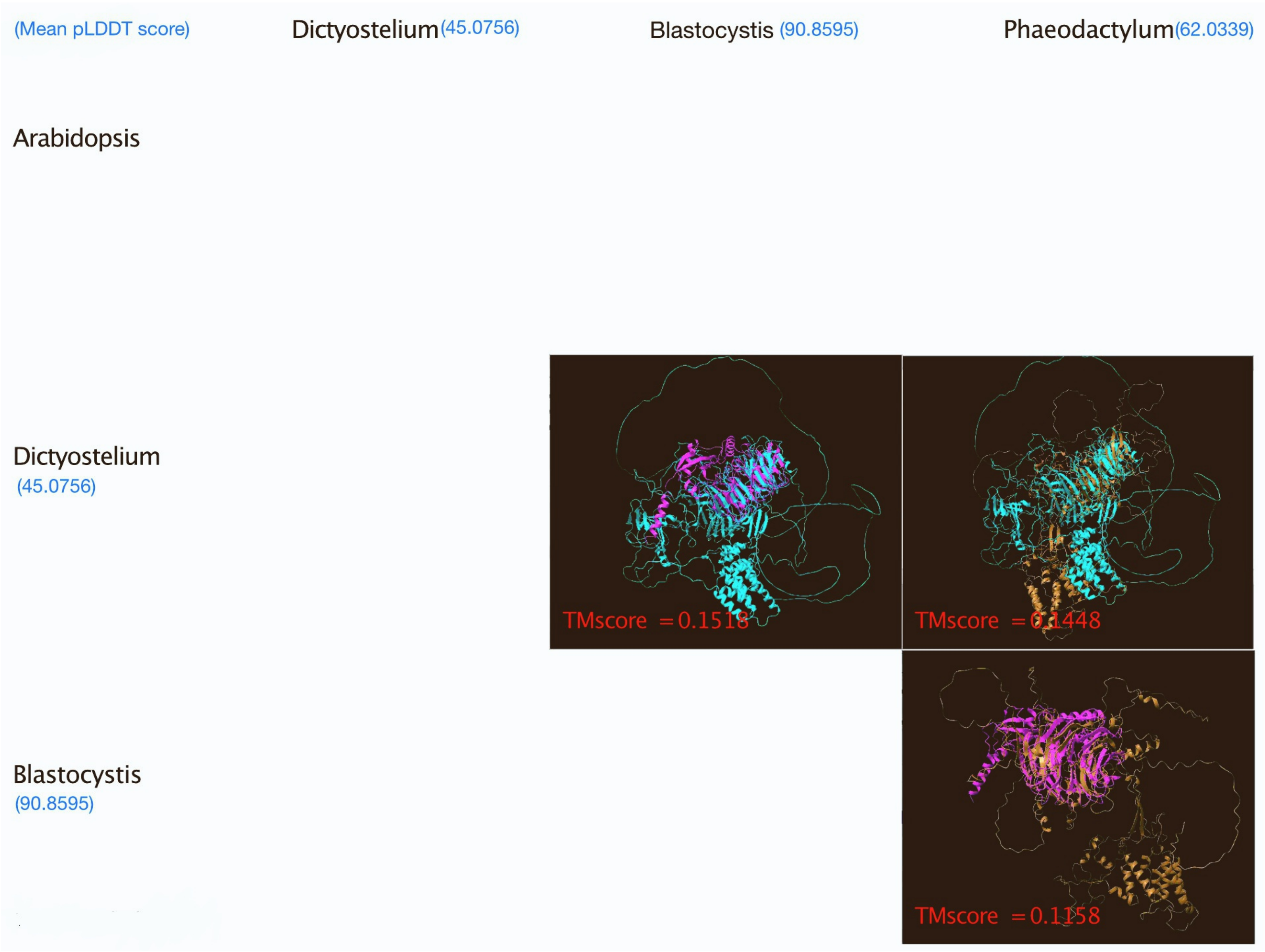

Data S4F: TM Align comparisons of AlphaFold predicted structures from all TSET subunits

All subunits from the TSET complex were predicted and aligned between all pairwise combinations of orthologues from *A. thaliana*, *D. discoideum*, *Blastocystis* sp., and *P. tricornutum*. TM scores are presented as normalised to the structure from the organism in the row. This gives two support values for each structural relationship, providing additional points for understanding structural similarity. Both TM scores of a relationship surpassing the 0.5 score threshold would indicate strong structural similarity. pLDDT scores for each predicted structure are provided, with a score >70 indicating confidence in the prediction, and >90 indicating high confidence. A: TSPOON. B. TSAUCER. C: TPLATE. D: TRAY1. E: TCUP. F: TTRAY2. G: PAE plots for all predicted subunits. Structural models shown in Figure 3 are highlighted in yellow, with the PAE graphs showing relatively reliable predictions, consistent with their TM scores near or above 0.5. Related to Figure 3.

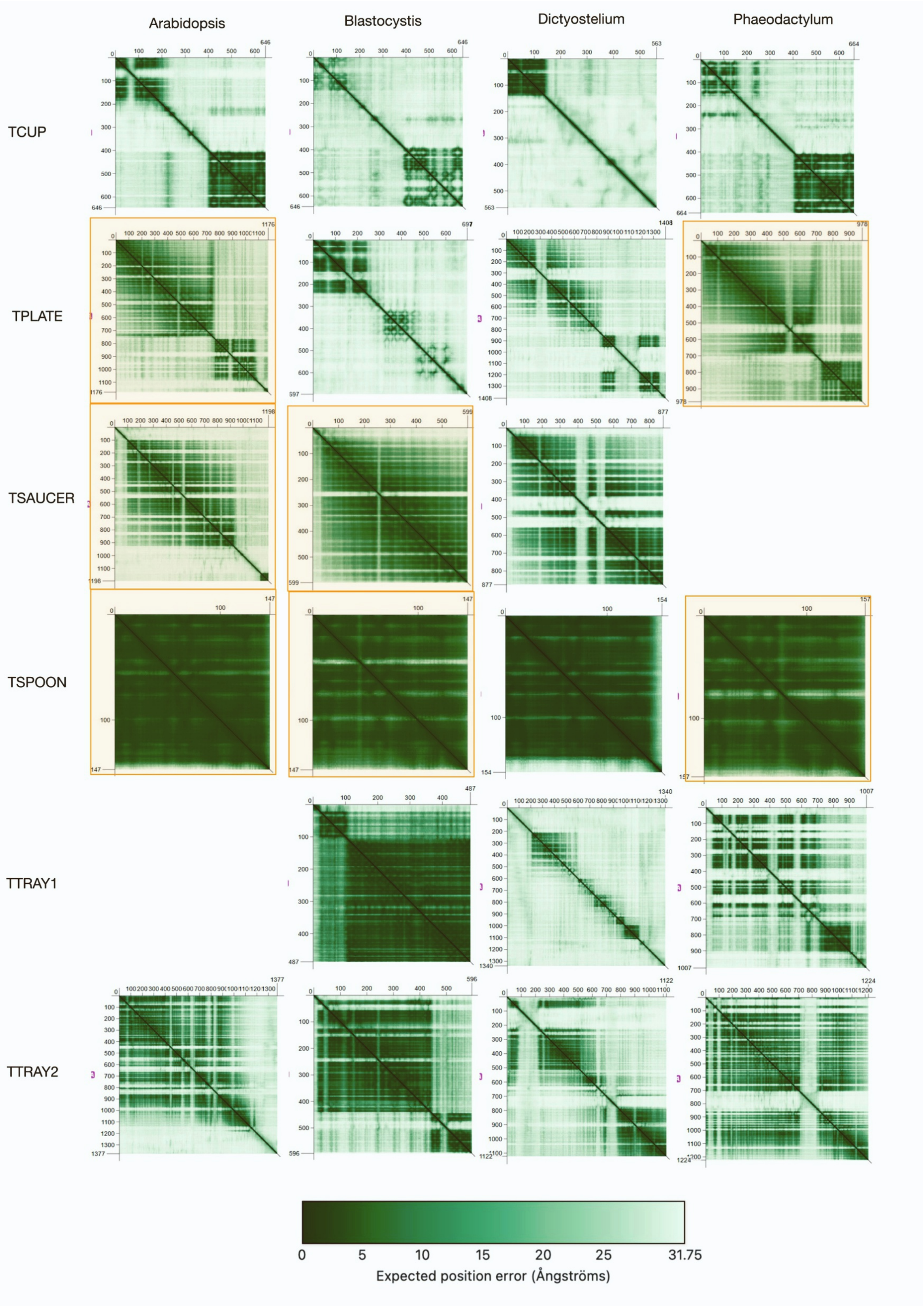

Data S4G: TM Align comparisons of AlphaFold predicted structures from all TSET subunits

All subunits from the TSET complex were predicted and aligned between all pairwise combinations of orthologues from *A. thaliana*, *D. discoideum*, *Blastocystis* sp., and *P. tricornutum*. TM scores are presented as normalised to the structure from the organism in the row. This gives two support values for each structural relationship, providing additional points for understanding structural similarity. Both TM scores of a relationship surpassing the 0.5 score threshold would indicate strong structural similarity. pLDDT scores for each predicted structure are provided, with a score >70 indicating confidence in the prediction, and >90 indicating high confidence. A: TSPOON. B. TSAUCER. C: TPLATE. D: TRAY1. E: TCUP. F: TTRAY2. G: PAE plots for all predicted subunits. Structural models shown in Figure 3 are highlighted in yellow, with the PAE graphs showing relatively reliable predictions, consistent with their TM scores near or above 0.5. Related to Figure 3.

Data S5: Subunit docking modelling of TSET subunits.

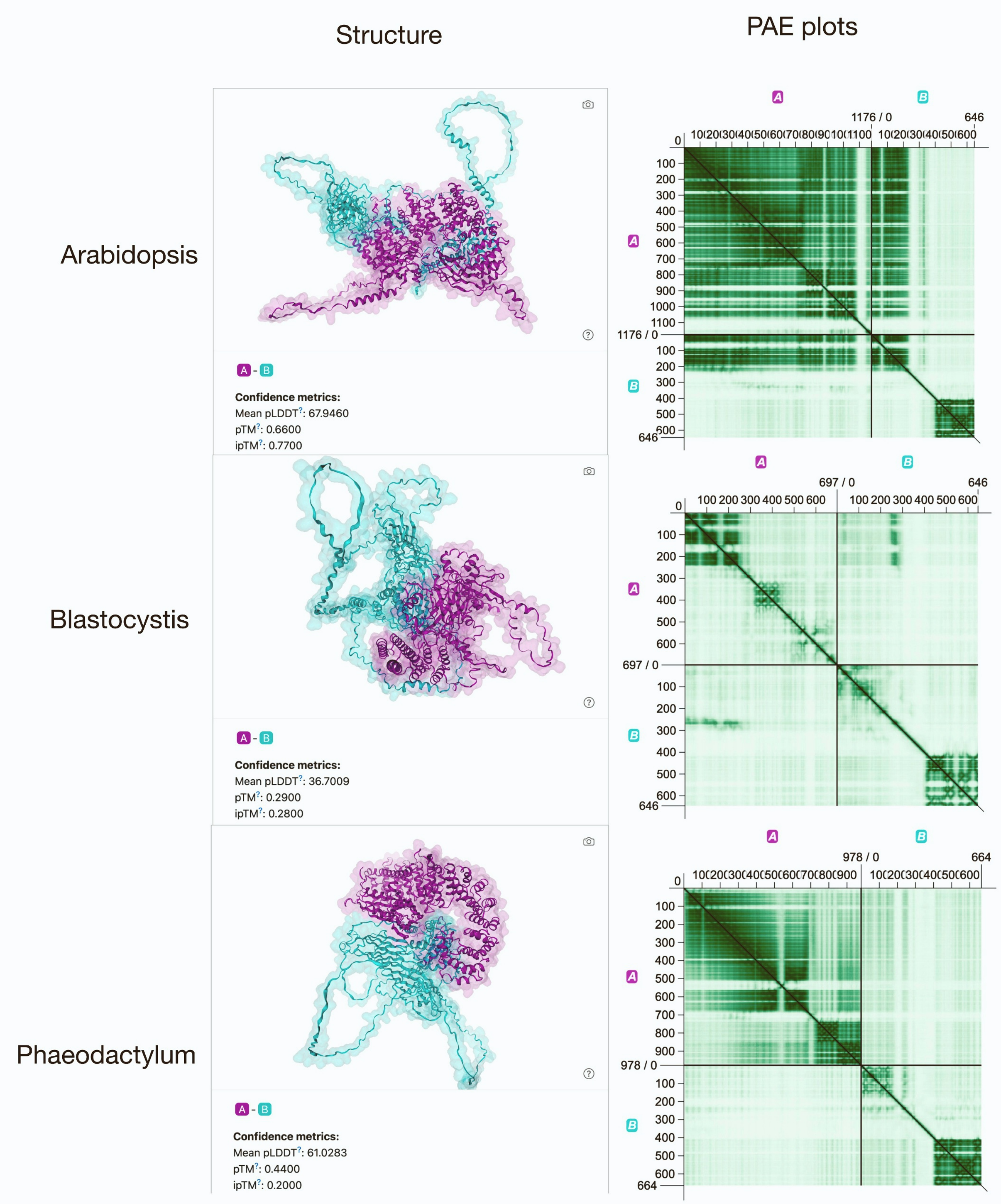

Data S5A: Subunit docking modelling of TSET subunits

A) For each of the pairs of TPLATE and TCUP from *A. thaliana*, *Blastocystis* sp., and *P. tricornutum*, the structural models were predicted showing individual structures and potential interactions. Predicted structures are shown to the left, PAE plots shown to the right. pLDDT scores for each predicted structure are near, but below, the confidence threshold (>70), in each case. Therefore we hesitate to draw conclusions from these analyses. B) TM scores for comparisons of the orthologous subunit-pair predicted structures between the respective taxa assessed. Although these scores are below the threshold of 0.5, the models of TSAUCER and TSPOON (Figure 4) are individually relatively confidently predicted. Related to Figure 4.

TSPOON-TSAUCER TMscores (normalized to the row)

	Arabidopsis	Blastocystis	Dictyostelium
Arabidopsis	-	0.2331	0.5761
Blastocystis	0.1661	-	0.1862
Dictyostelium	0.2845	0.2227	-

TPLATE-TCUP TMscores(normalized to the row)

	Arabidopsis	Blastocystis	Phaeodactylum
Arabidopsis	-	0.1671	0.2352
Blastocystis	0.1409	-	0.1492
Phaeodactylum	0.2204	0.1658	-

Data S5B: Subunit docking modelling of TSET subunits

A) For each of the pairs of TPLATE and TCUP from *A. thaliana*, *Blastocystis* sp., and *P. tricornutum*, the structural models were predicted showing individual structures and potential interactions. Predicted structures are shown to the left, PAE plots shown to the right. pLDDT scores for each predicted structure are near, but below, the confidence threshold (>70), in each case. Therefore we hesitate to draw conclusions from these analyses. B) TM scores for comparisons of the orthologous subunit-pair predicted structures between the respective taxa assessed. Although these scores are below the threshold of 0.5, the models of TSAUCER and TSPOON (Figure 4) are individually relatively confidently predicted. Related to Figure 4.
