# Supplementary figures and images for: Prevalence and environmental abundance of the TSET complex in cosmopolitan algal groups
Source: iScience. 2025 May 15;28(6):112679. doi: 10.1016/j.isci.2025.112679 (PMC12167817; doi:10.1016/j.isci.2025.112679)

TCUP

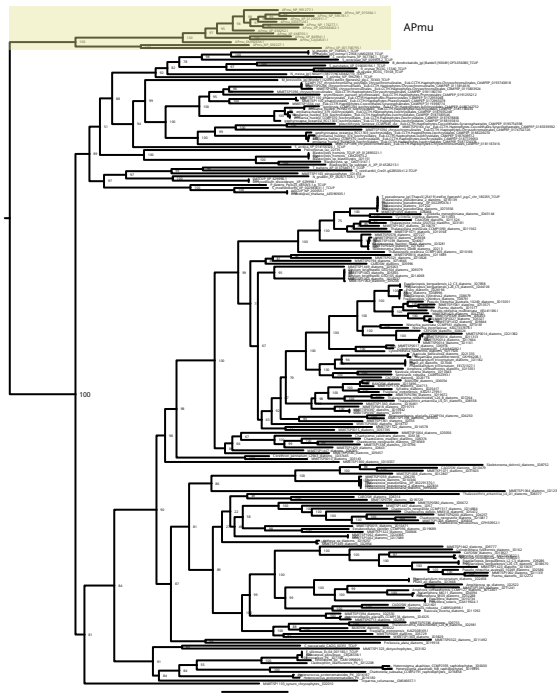

TPLATE

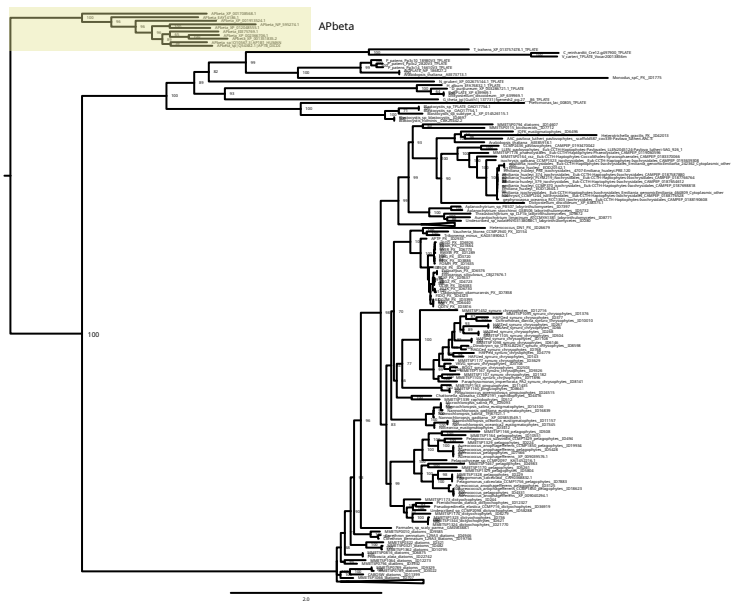

TSPOON

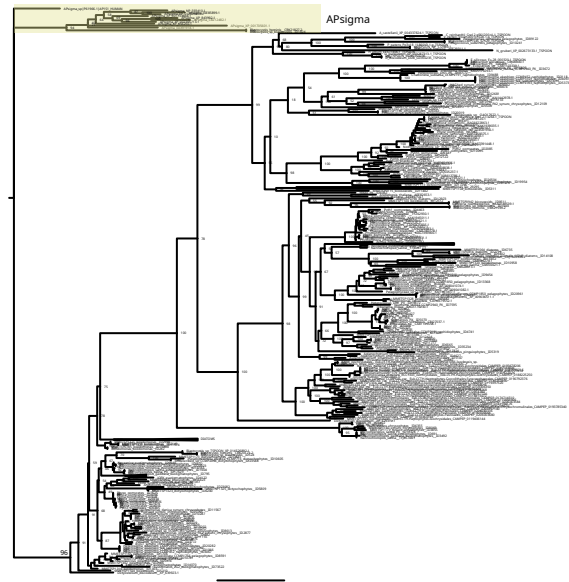

TSAUCER

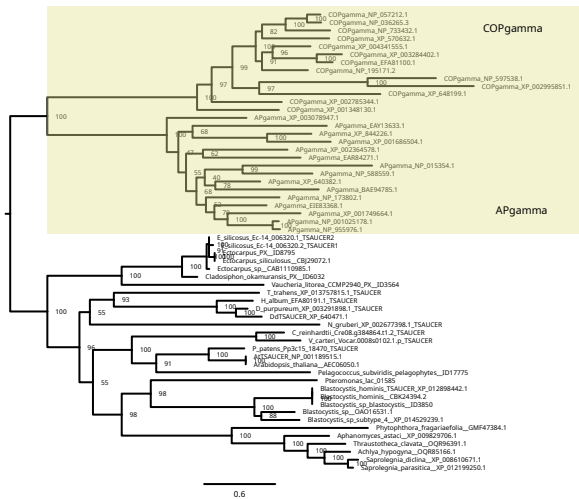

TTRAY1

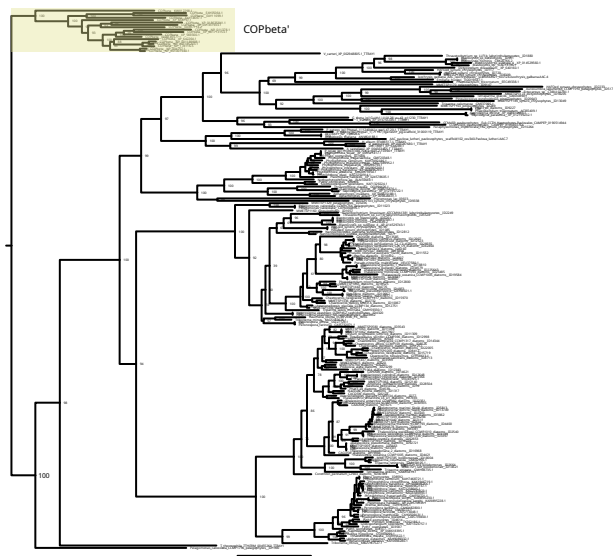

TTRAY2

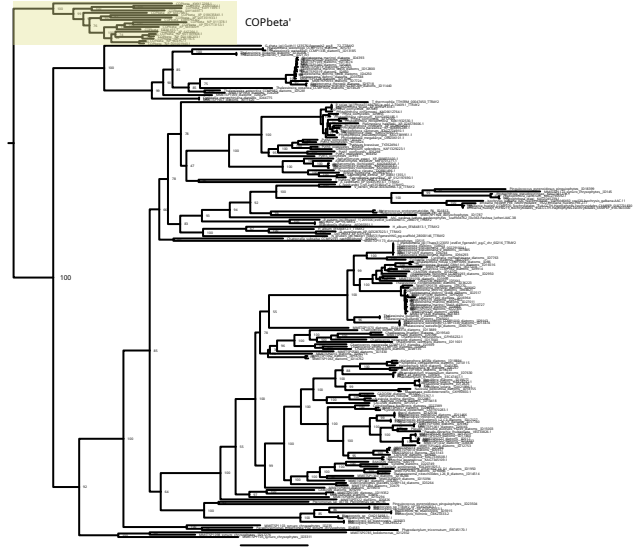

Supplement: Data S3. Datafiles used in informatic analyses, related to Figures 1 and 2 — List of TSET homologues, sequences of query TSET proteins, alignments and curated tree topologies, and Ref_seqs_1_manual_predictions.csv used for the AMOEBAE searches. [file mmc4.zip › S3_files/Trees_hapto_stram/Trees.pdf]
